# Associations between Knowledge of the Causes and Perceived Impacts of Climate Change: A Cross-Sectional Survey of Medical, Public Health and Nursing Students in Universities in China

**DOI:** 10.3390/ijerph15122650

**Published:** 2018-11-26

**Authors:** Lianping Yang, Wenmin Liao, Chaojie Liu, Na Zhang, Shuang Zhong, Cunrui Huang

**Affiliations:** 1 School of Public Heath, Sun Yat-sen University, Guangzhou 510080, China; yanglp7@mail.sysu.edu.cn (L.Y.); liaowm3@mail2.sysu.edu.cn (W.L.); zhangn23@mail2.sysu.edu.cn (N.Z.); 2 School of Psychology and Public Health, La Trobe University, Melbourne 3086, Australia; c.liu@latrobe.edu.au; 3 Center for Chinese Public Administration Research, School of Government, Sun Yat-sen University, Guangzhou 510275, China

**Keywords:** climate change, health impact, knowledge, perception, university students

## Abstract

This study aimed to measure the knowledge and perceptions of medical, public health, and nursing students about climate change and its impacts, and to identify associations between the knowledge and perceptions. Data were from a nationwide cross-sectional survey of 1387 students sampled in five different regional universities in China (April–May 2017). The knowledge and perceptions of the participants were collected by self-administered questionnaires. We found that most respondents believed that climate change is generally “bad” (83%) and bad for human health (88%), while 67% believed that climate change is controllable. The vast majority of respondents acknowledged illness conditions resulting from poor air quality (95%), heat stress (93%), and extreme weather events (91%) as potential impacts of climate change. Nevertheless, only 39% recognized malnutrition as a consequence of food deprivation resulting from climate change. Around 58% of respondents could correctly identify the causes of climate change. The knowledge of the causes of climate change was not associated with the ability to recognize the health consequences of climate change. However, the knowledge of causes of climate change was a significant predictor of increased awareness of the negative impacts of climate change between the medical and nursing students, although this was not the case among their public health counterparts. Poor knowledge about the causes of climate change is evident among students in China. They are able to recognize the direct links between weather events and health, but less likely to understand the consequences involving complicated pathways. Research and training into the underlying mechanisms of health impacts of climate change needs to be strengthened.

## 1. Introduction

Climate change is imposing one of the biggest global health threats in 21st century [1,2,3]. A lack of adequate response to climate change can undermine the last half century of gains in global health [1]. The health impacts of climate change can be a direct effect of weather events, such as heat stress, floods, drought and storms, and/or an indirect effect as a consequence of displacement and mental disorders, air pollution, spread of disease vectors, food insecurity, and malnutrition [1,4,5].

Health professionals are supposed to play a critical role in mitigating the health risks of climate change [6,7]. Physicians, nurses, public health specialists, and health officials should be equipped with high expertise and take essential responsibilities in protecting populations from the harm of climate change [8]. Millions of patients all over the world seek consultations and treatments from physicians every day. Physicians also enjoy a high level of recognition and trust in the society. They are well positioned to exert influence on the society for the common good, advocating for environment-friendly lifestyles and pro-health public policies [9]. Nurses are bound by both clinical practices and professional ethics to address concerns of their patients and prevent adverse health outcomes [10]. Public health specialists have endeavored to have a broad view on population health. They can support the implementation of programs and policies in ways that enhance physical and social environments at the local, national, and international levels [8]. Therefore, the preparedness of health professionals forms a critical part of the global response to climate change. 

University education is a good starting point for preparing health professionals. Mitigation and adaptation strategies in response to climate change can be taught in universities under the umbrella of sustainability. Higher education has been widely considered to be critical for the sustainable development of the world [11,12,13]. Sustainability-related issues, like environmental pollution, climate change, overconsumption, and exploitation of resources are often addressed through various curricula. Zsoka et al. [14] explored the impacts of environmental education on the environmental knowledge, attitudes, and “greening” daily activities of high school and university students, and found out that the focus of environmental education is essential in shaping the attitudes of the students toward sustainable consumptions. But it remains a great challenge in developing proper strategies for priming medical students into a “medical mode” that can alter their opinions on the scientific merit of nonmedical issues [15]. Ng et al. highlighted the importance of emphasizing the ethical aspects of corporate social responsibility in undergraduate education programs for the future professional accounts [16]. However, there is a shortage of research documenting effective ways of teaching about sustainability in universities. University students are still forming their values and beliefs. A one-fits-for-all approach in acquiring sustainability competences is not feasible [17]. Environmental education for students in health-related disciplines, for example, has to consider the specific contexts of their professions. Without proper education, they may hold naïve awareness of the potential impact of individual contributions and feel disempowered for actively engaging in sustainability efforts [11]. Chaplin and Wyton pointed out the importance of understanding and addressing the value-action gap in motivating university students to engage with sustainability [12]. Kagawa suggested that future studies should be directed towards identifying various means of facilitating pro-sustainability behaviors [18]. 

Knowledge is a key driver of global actions on climate change [19]. Empirical evidence shows that the public concern about climate change is shaped by their acquired knowledge [19,20,21]. Climate-relevant knowledge appeared important for the public’s willingness to change behaviors and to accept climate change policies [21]. However, not all dimensions of knowledge are equally influential. Knowledge about the causes of climate change is perhaps the most powerful driver of concerns about and actions on climate change, although it has been subject to serious debates over the past few decades in the public and political arenas [22,23]. A cross-country survey conducted by Shi and colleagues revealed that a higher level of concerns from the general public on climate change is associated with their understanding on the causes of climate change [19]. Bord et al. found that causal knowledge of global warming is associated with beliefs and behavioral intentions about climate change [24]. Similar results were also found in a study that was conducted by Tobler et al. [25]. Based on these findings, Shi and colleagues argued that climate change campaigns should have a strong focus on promoting knowledge about the causes of climate change [21].

There is paucity in the literature documenting the importance of causal knowledge on raising awareness and competency of health workers in response to the health impacts of climate change. Although medical, nursing, and public health students are likely to engage in or be exposed to climate change debates, little is known about how these students incorporate climate change knowledge into their professional learning. There has been an increasing call for teaching about climate change in medical education [26]. Some medical schools, such as those in the USA, have indeed started to offer coursework on “climate change and health”. However, it is by no means arranged as a standard requirement [27]. There is a lack of consensus about what should be taught about climate change in medical, nursing, and public health curricula.

This study aimed to fill the literature gap by conducting a survey in a representative sample of medical, nursing, and public health students in China. The preparedness of health workforce in actions on climate change is particularly important for China, because China is highly vulnerable to global climate change due to its large population size and rapid transition in economic and social development. Findings of this study may help educational providers to better incorporate climate change components into their teaching deliveries.

## 2. Materials and Methods

A nationwide cross-sectional questionnaire survey was conducted on medical, nursing, and public health students in a representative sample in China during April and May in 2017.

### 2.1. Study Participants

Senior university students majoring in clinical medicine, nursing, and public health were eligible to participate in this study. The participants were restricted to those who had one more year to finish their undergraduate studies: year four in clinical medicine and public health; and, year three in nursing. These students had already completed all required coursework and were ready for the final year practice placements. Their completed coursework usually contained two parts: half in relation to basic biomedicine subjects and half in relation to clinical/professional subjects [28].

A multistage stratified cluster sampling method was adopted to recruit participants. China has been officially divided into five geographic regions. One university in each region was identified: Harbin Medical University in the north, Sichuan University in the west, Sun Yat-sen University in the south, Fujian Medical University in the east, and Huazhong University of Science and Technology in the central region. We estimated that a minimal sample size of 250 from each participating university would be able to meet the requirements of sub-group statistical tests and regression analyses: 110 in clinical medicine; 80 in public health; and, 60 in nursing in line with the proportion of enrolled students for each degree program. Students in these participating universities studied in groups from beginning to completion, each comprising 30–40 members. These groups formed the basic unit for sampling. We randomly selected two or three groups of students for each major in each participating university and invited all of the group members to participate in the survey.

### 2.2. Data Collection

A questionnaire was developed based on the theories of KAP (knowledge, attitudes, and practice) and rational choice action. These theories hypothesize that individuals may choose a particular course of action that is in line with their beliefs and preferences, and knowledge plays a crucial role in shaping these beliefs and preferences [12,29,30]. Human beings are characterized by pursuing the causal structure of the world that supports cognitive achievements [31]. Causal knowledge plays a central role in the human thoughts [32]. It allows people to provide an explanation on the occurrence of an event and to predict the future events and even the consequences of counterfactuals. This forms the foundation of reasoning and rational actions.

The questionnaire comprised three sections, with items deriving from the existing literature [10,19,33,34,35,36] (Table S1). Section One tapped into the general perceptions of the respondents on the nature and impacts of climate change. Example questions included “whether climate change is controllable” and “whether climate change is bad for human health”. Respondents were asked to rate their opinions on a six point Likert scale without a neutral midpoint. Such an approach was recommended by Maibach et al. to better capture the inclination of the participants [37]. Section Two asked the respondents to identify the health impacts of climate change from a list of 12 health-related problems. These 12 aspects of problems have been widely accepted as potential important health impacts of climate change [10,34]. Section Three investigated the knowledge of respondents about the causes of climate change. A list of four statements [19] were presented and the respondents were asked to judge whether they are true or false. Example statements included “Climate change is mainly caused by human activities” and “The global CO_2_ concentration in the atmosphere has increased during the past 250 years”.

The questionnaires were distributed by our trained investigators to the classrooms of the selected groups of students. They were asked to complete the questionnaires independently and returned the questionnaires to a collection box when they left the classrooms. A total of 1436 questionnaires were disseminated and 1387 (response rate, 97%) were returned and valid for data analyses. 

### 2.3. Data Analyses

Perceptions on the nature and impacts of climate change were recoded into binary data (“yes vs. no” or “correct vs. incorrect”) for the purpose of data analyses. The percentage of positive or correct responses in relation to each item was compared between different streams of students using χ^2^ tests.

The ability of respondents to identify the health impacts of climate change was assessed using the percentage of correct answers related to the 12 listed problems, which was further categorized into high (≥50%) and low (<50%) levels. We also calculated a summed score (0–12), reflecting the total number of problems that were correctly identified by the respondents. This served as an indication of the overall ability of an individual to identify climate change related health problems.

Similarly, knowledge of the causes of climate change was assessed using the percentage of correct answers related to the four tested statements, which was further categorized into high (≥50%) and low (<50%) levels. A summed score (0–4) was calculated to reflect the overall understanding of an individual on the causes of climate change.

Multivariate logistic regression models were established to examine the associations between knowledge of the causes of climate change (high vs. low) and perceived impacts of climate change (“yes or no” on seven questions), controlling for variations in social demographic characteristics (gender, age, location, household income, and health status). Because multiple indicators (perceived impacts of climate change as dependent variables) were used in the logistic regression tests, we used the Bonferroni procedure to adjust the *p* values in regard to the statistical significance of the adjusted odds ratios (AORs) of causes knowledge [38]. We also performed sensitivity tests by introducing the summed score (0–4) of causes of knowledge into the regression models.

## 3. Results

### 3.1. Characteristics of Participants

Of the 1387 respondents, 69% were 21–22 years old and 67% were female. The majority (>80%) rated their health status as good or very good. The nursing students were more likely to be female (*p* < 0.01) and reported lower household income (*p* < 0.01) (Table 1).

### 3.2. Perceived Impacts of Climate Change

About 67% of respondents believed that climate change is controllable, as compared with 13% who believed it is uncontrollable. The majority of respondents (>80%) agreed that climate change has negative impacts on the public in general and for human health specifically. Over 85% of respondents anticipated serious health impacts of climate change in the next 20 years at the local, national, and international levels. These perceptions were consistent across different streams of students, except that the public health students were more likely (*p* < 0.01) to believe climate change is controllable (Table 2).

Overall, the respondents were able to identify on average 9.5 problems (SD 2.3) out of a total of 12 related to climate change. The vast majority of respondents acknowledged illness conditions and service disruptions resulting from poor air quality (95%), heat stress (93%), and extreme weather events (91%) as potential health impacts of climate change. Nonetheless, only 39% recognized malnutrition and 64% recognized mental health illness as a consequence of climate change. About 20–30% of respondents believed that the list of problems were exhaustive. The medical students were more likely to be able to recognize water-borne infectious disease, food-borne infectious disease, mental health illness, and malnutrition as a consequence of climate change as compared to their nursing and public health counterparts (Table 2).

### 3.3. Knowledge about the Causes of Climate Change

Overall, the respondents achieved a mean score of 2.31 and 58% correctly identified all of the answers about the causes of climate change. About 53% medical students, 50% public health students, and 41% nursing students achieved a high level of knowledge about the causes of climate change: the differences appeared to be statistically significant (χ^2^ = 11.06, *p* < 0.01). 

Most respondents accepted the general truth of increasing earth temperature (64%), increasing CO_2_ in the atmosphere (75%), and the contribution of human activities to climate change (77%). However, only 16% of respondents were able to identify details about the change in CO_2_ concentrations over the past few centuries (Table 3).

### 3.4. Associations between Knowledge on the Causes and Perceived Impacts of Climate Change

The logistic regression models showed that there was no significant association between the knowledge on the causes of climate change and the ability of the respondents to recognize climate change related health problems (Table 4).

However, a higher level of knowledge about the causes of climate change was a significant predictor of increased awareness of the negative impacts of climate change, in particular among the medical students (with an AOR ranging from 1.83 for “climate change is controllable” to 9.18 for “health impacts of climate change will be serious in China”). However, among the public health students, knowledge on the causes of climate change did not appear as a significant predictor of increased awareness on the negative impacts of climate change. Among the nursing students, the knowledge level was only a significant predictor of the overall perception on the negative impacts of climate change. The sensitivity tests produced consistent results, although the AORs changed (Tables S31–S34). 

## 4. Discussion

There was an overwhelming belief in the respondents that climate change is generally “bad” and bad for human health. However, only 60% of the respondents could correctly identify the causes of climate change. On average, the respondents identified nine health problems that were related to climate change out of a total of 12 in the list. Knowledge on the causes of climate change is not associated with the ability to recognize the health consequences of climate change. Nevertheless, it is a significant predictor of increased awareness of the medical and nursing students on the overall negative impacts of climate change.

Higher education can and should play an important role in raising awareness and shaping the attitudes and practices of the new generations toward actions on sustainability. The values and behaviors of university students with the sustainability agenda are important within the climate change context. There are some university initiatives that are known as greening the curriculum [39]. Chaplin and Wyton found that university students tend to accept the importance of sustainable living, yet their understanding is usually at a very low level and there is a shortage of actions [12]. It is critical to close the gap between knowledge, values, and actions [11].

### 4.1. Perceptions about Climate Change and Its Health Impacts

Overall, perceptions on the impacts of climate change are consistent across the three streams of students. They all anticipated increasing consequences of climate change in the near future at the local, national, and international levels. However, the immediate health challenges that are associated with extreme weather events (e.g., heat stress and service disruptions) are more likely to attract their attentions when compared with those indirect, more complex, and long lasting health consequences (e.g., changing ecosystem).

It appears that it is less likely that these students would adopt a global view on the impacts of climate change. Mental health conditions and malnutrition, which are often a result from population displacement (e.g., climate refugees), came to the bottom on the list of recognized health challenges that are associated with climate change. This phenomenon is not unique in China. People’s concerns are naturally connected with their past experiences and the environment in which they grow up. Empirical evidence shows that the world is experiencing increasing catastrophic weather events over the past few decades. However, these events appear in various forms in different locations [1]. Air quality related illness is the most recognizable health problem in this study, perhaps because air pollution has been an extraordinary environmental problem in China [40]. By contrast, in Ethiopia, health science students reported flooding, drought, and increasing temperature as the most prominent impacts of climate change [41]. In the USA, local health officers considered extreme hot weather and wildfire as the most serious threat of climate change [34].

The medical and nursing students in this study demonstrated a lower level of confidence on the prospect of controlling climate change than their public health counterparts. This may be a reflection of the limitations of their professional training: medical and nursing students are usually better equipped with skills for individual actions than those for collective actions [18]. The weak confidence on collective actions may eventually jeopardize the ability of medical and nursing professionals to play an imperative role in the debate of and actions on climate change [42]. On the other hand, we found that the public health students were less likely to be able to identify water-borne infectious disease, food-borne infectious disease, mental health conditions, and malnutrition as a consequence of climate change as compared with their medical and nursing counterparts. This is not surprising given the recent trend of public health education, shifting from a focus on disease prevention to a focus on health promotion. Public health professionals are becoming more interested in social, environmental and behavioral determinants of health, which are usually associated with multiple disease conditions. Internationally, there are increasing numbers of public health professionals without a qualification of biomedical training [10,34].

### 4.2. Low Level of Knowledge on the Causes of Climate Change

Overall, the students demonstrated a low level of knowledge about the causes of climate change, with 58% giving correct answers to all of the four knowledge questions as compared with an average of 60% in the general public revealed in a cross-country study [19]. The nursing students had a lower level of knowledge than their medical and public health counterparts. It is not clear whether this is caused by the differences in educational programs or the differentiations in professional functions and cultures. 

There has been a strong consensus in the scientific community that global warming has increased significantly in the last century due to human activities [43]. Nonetheless, we found a significant level of disagreement in the student respondents with the scientific conclusion: more than 35% disagreement on global warming and more than 25% disagreement on CO_2_ changes in the past 250 years. Internationally, there are around 17% of the public respondents (age over 20) that do not think the CO_2_ concentration changed and 36% that do not perceive the temperature increase [19]. Therefore, university students did not show higher correct consensus when compared to the general public. 

### 4.3. Higher Level of Knowledge on the Causes of Climate Change Is Associated with Higher Awareness of the Negative Impacts of Climate Change

Some researchers argued that a good understanding about the causes of climate change is essential for motivating health professionals to embrace eco-friendly practices and advocate for green and healthy lifestyles: they have to believe in human causes to climate change [43,44]. In Pakistan, the public perception on climate change is shaped by their knowledge relating to heat waves within a climate change context [45]. Medical students should be educated to endorse the importance of containing CO_2_ emissions so that they can develop confidence and be empowered to play an active role in global actions on climate change [1,27]. Indeed, a higher level of knowledge on the causes of climate change is a significant predictor of increased awareness on the negative impacts of climate change. This is particularly true for medical students, as revealed in this study. Knowledge and perceptions are usually considered as prerequisites for the general public to adapt to climate change [46].

However, knowledge on the causes of climate change by itself is not enough to enhance the skills of medical, public health, and nursing students in recognizing and mitigating health harms resulting from climate change. We found that knowledge on the causes of climate change is not associated with the ability of the students to recognize climate change related health problems. Kahan and colleagues [47] also found that knowledge has limited effects on people’s perceptions on climate change, let alone behavioral changes. Grimm [31] concluded that causal knowledge can lead to skill gains, but only under certain conditions. Hagmayer and Witteman proposed a Causal-explanation-based Decision Making (CDM) framework, which describes when and how causal knowledge and reasoning should be used in decision making for better outcomes [32]. They pointed out that the CDM framework aligns well with the clinical reasoning of health professionals. It is important to note that the causal knowledge about climate change measured in this study does not entail understandings about the underlining mechanisms of the potential health impacts of climate change. Our limited understanding on these mechanisms may jeopardize the effect of the causal knowledge on the skill gains of the students in recognizing climate change related health problem.

It appears that knowledge on the causes of climate change may have varied effects on different health professionals. In this study, we found that the knowledge on the causes of climate change has no significant associations with perceived impacts of climate change in public health students. However, it is a significant predictor of perceived negative impacts of climate change in medical students, as is consistent with findings of a study on the general public across six countries [19]. Since different health professionals play a different role in climate change actions, some researchers have called for developing different approaches, such as teaching, learning, and assessment approaches in integrating climate change topics into the teaching curricula for medical, nursing, and public health students [27,48,49].

### 4.4. Implications on Teaching and Research into Health Impacts of Climate Change

Health professionals can play various roles at different levels in global actions on climate change [50]. At the individual level, they identify the patients and those who are in high risks of developing health problems as a consequence of climate change. They take consideration of the environmental factors in treating and preventing relevant disease conditions. At the practice level, they need to be conscious of the environmental impacts of their business operations and engaged in reduction of CO_2_ footprints. At the population level, they are role models of the community and they advocate social and environmental actions for improving population health [50,51].

Obviously, different skill sets are required for competent performance of the above roles [48]. It is evident from this study that the students lack a global and systems view on health impacts of climate change despite high awareness of the negative impacts of climate change. They have difficulties in recognizing health problems that are indirectly linked to climate change through more complex pathways, including those that are associated with population displacement and changing ecosystems. In the literature, there have been discussions about how to reinforce knowledge on the interaction between climate change and human health in medical education [48]. However, such education programs have to adopt a global view. It is not ethical for health professionals to constrain services to the concerns of their local patients only, and ignore the broader problems that are experienced by those from the “other parts of the world” as a result of climate change [51]. Meanwhile, teaching into climate change has to be meaningful and bear close connections with the reality the health professionals are facing [26]. China is not a migrant country. The concept of climate refugee may sound alien to many health professionals in China. However, the recent transition of China’s economy has witnessed dramatic growth in population movements (e.g., rural-to-urban and region-to-region migrations) and changes in ecosystems (e.g., the three gorges dam). In recent years, China has also accelerated its international health assistance activities to other countries. As a result, health professionals in China have been increasingly exposed to many health challenges that are similar to some of the problems resulting from climate change. It would be a reasonable choice to combine the considerations of development and climate change in teaching deliveries [26].

No doubt, climate change should be integrated into the curricula for medical, public health, and nursing students [52]. Nevertheless, this is by no means a simple and straightforward task. There are a number of challenges: (1) Medical curricula have already been overcrowded [27]; (2) We need to apply different pedagogies in teaching for different health professionals [44]; (3) Our understanding on the mechanisms of health impacts of climate change is still limited; and, (4) There is a serious shortage of academic staff in medical, public health, and nursing schools who are competent in teaching and research into the area of climate change. At this stage, most “climate change” education programs have restricted contents to the trend and causes of climate change. Although this is important for raising awareness and building motivations for actions, it is far less than enough to empower health professionals to engage in actions on climate change. Research into the underlying mechanisms of health impacts of climate change needs to be strengthened. There is also a need to change the culture of professional practices: health professionals need to see themselves as an integral part of the global action on climate change. Environmental education is important [53,54], but it needs to be integrated into the professional education programs as a higher order of learning beyond the confines of professional knowledge and skills [16]. Teaching about climate change typically takes a wider sustainability focus [18,48]. The Higher Education Academy (HEA) in the UK developed some examples about skills and knowledge that are necessary for an “action-oriented, sustainability literate” graduate [18].

To our knowledge, this is the first national study to explore the knowledge and perceptions on climate change of medical, public health and nursing students in China. Similar studies are also limited in the international literature. There are some limitations in this study. The study instrument was developed based on the few available literature. The reliability and validity of the instrument are subject to further scrutiny. The study adopted a cross-sectional design. No causal relationships can be drawn from the results. In addition, perceptions can be shaped by many factors, including culture, values and religious belief, just to name a few. Unfortunately, many of these data were either unavailable or hard to measure due to a lack of reliable measurement instruments. Furthermore, this study was undertaken in China. Precautions should be taken in attempts to generalize the findings to other countries. However, China’s medical education system has borrowed experiences from many other countries. It has become increasingly aligned with international standards. Lobell et al. point out that Asia and Africa are likely to suffer similar problems due to the same likelihood of large food-insecure human populations [55]. Future studies are warranted to explore the effective ways of incorporating climate change contents into medical curricula. There is also a need to develop a more comprehensive instrument to assess competencies of health professionals in dealing with climate change and its health impacts.

## 5. Conclusions

Medical, public health, and nursing students in China are highly aware of the negative impacts of climate change; but, it is evident that they have poor knowledge about the causes of climate change. The students are able to recognize the direct links between weather events and health; but, they are less likely to be able to understand the consequences of climate change involving complicated pathways. A higher level of knowledge on the causes of climate change is a predictor of increased awareness on the negative impacts of climate change; but, the ability to recognize health consequences of climate change is not associated with knowledge on the causes of climate change. Although a good understanding on the causes of climate change by itself is important, it is not enough to get health professionals prepared to cope with climate change consequences. Training and research into the underlying mechanisms of health impacts of climate change needs to be strengthened.

## Figures and Tables

**Table 1:** Demographics of participants by different majors.

Characteristics	All (*n* = 1387)		Medical (*n* = 644)		Public Health (*n* = 430)		Nursing (*n* = 313)	
	*n*	(%)	*n*	(%)	*n*	(%)	*n*	(%)
Region								
East	258	18.6	117	18.2	80	18.6	61	19.5
North	308	22.2	156	24.2	79	18.4	73	23.3
Central	248	17.9	107	16.6	84	19.5	57	18.2
West	262	18.9	120	18.6	83	19.3	59	18.9
South	311	22.4	144	22.4	104	24.2	63	20.1
Gender								
Female	926	66.8	328	50.9	319	74.2	279	89.1
Male	461	33.2	316	49.1	111	25.8	34	10.9
Age								
≤20	126	9.5	38	6.1	20	5.0	68	23.1
21–22	913	68.9	419	66.7	302	74.8	192	65.3
>22	287	21.6	171	27.2	82	20.3	34	11.6
Monthly household income per capita (¥)								
<1000	154	11.2	62	9.8	51	12.0	41	13.1
1000–1999	284	20.7	112	17.6	95	22.4	77	24.6
2000–4999	514	37.5	239	37.6	155	36.6	120	38.3
5000–9999	293	21.4	146	23.0	89	21.0	58	18.5
≥10,000	127	9.3	76	12.0	34	8.0	17	5.4
Self-rated health								
Very good	303	21.9	156	24.2	80	18.6	67	21.4
Good	858	61.9	378	58.7	275	64.0	205	65.5
Medium or poor	226	16.3	110	17.1	75	17.4	41	13.1

**Table 2:** Perceived impacts of climate change from medical, public health and nursing students.

Perceived Impacts of Climate Change	Medical (*n* = 644)		Public Health (*n* = 430)		Nursing (*n* = 313)		All (*n* = 1387)		*p*
	*n*	(%)	*n*	(%)	*n*	(%)	*n*	(%)	
Climate change is controllable	418	64.9	321	74.7	196	62.6	935	67.4	<0.01
Overall, climate change is bad	546	84.8	359	83.5	253	80.8	1158	83.5	0.30
Climate change is bad for human health	575	89.3	381	88.6	266	85.0	1222	88.1	0.15
Climate change will be serious in my local community	576	89.4	368	85.6	282	90.1	1226	88.4	0.09
Climate change will be serious in China	619	96.1	413	96.1	298	95.2	1330	95.9	0.79
Climate change will be serious in the world	609	94.6	410	95.4	299	95.5	1318	95.0	0.76
Health consequences of climate change									
Air quality-related illness	604	93.8	413	96.1	299	95.5	1316	94.9	0.22
Heat-related illness	596	92.6	407	94.9	283	90.4	1286	92.8	0.07
Disruption of health services by extreme weather events	585	90.8	396	92.1	282	90.1	1263	91.1	0.62
Cold-related illness	570	88.5	385	89.5	276	88.2	1231	88.8	0.82
Flooding-related displacement	550	85.5	375	87.4	259	82.8	1184	85.5	0.20
Illness related with shortage of water supply	556	86.5	352	82.2	269	86.2	1177	85.1	0.13
Vector-borne infectious disease	541	84.0	359	83.9	249	79.8	1149	83.0	0.23
Water-borne infectious disease	544	84.6	328	76.6	249	79.6	1121	81.0	<0.01
Other health impacts of climatic change	479	76.4	348	82.1	234	76.0	1061	78.1	0.06
Food-borne disease	472	73.4	250	58.1	226	72.2	948	68.4	<0.01
Mental health conditions	443	68.8	255	59.3	185	59.1	883	63.7	<0.01
Malnutrition	282	43.9	141	32.8	117	37.4	540	39.0	<0.01
Total score (Mean ± SD)	9.70 ± 2.39		9.36 ± 2.20		9.36 ± 2.27		9.52 ± 2.31		0.03

**Table 3:** Correct answers on the causes of climate change from medical, public health, and nursing students.

Causes of Climate Change	All		Medical		Public Health		Nursing		*p*
	*n*	%	*n*	%	*n*	%	*n*	%	
Climate change is mainly caused by human activities	1071	77.3	508	78.9	333	77.4	230	73.7	0.17
The global CO_2_ concentration in the atmosphere has increased during the past 250 years	1034	74.7	484	75.2	321	75.0	229	73.2	0.80
The last century’s global increase in temperature was the largest during the past 1000 years	880	63.6	420	65.2	285	66.4	175	56.5	0.01
Today’s global CO_2_ concentration in the atmosphere has already occurred in the past 650,000 years	217	15.7	94	14.6	75	17.5	48	15.4	0.45
Total score (Mean ± SD)	2.31 ± 1.01		2.34 ± 1.02		2.36 ± 1.01		2.18 ± 0.98		0.02

**Table 4:** Associations between understandings of the causes of climate change and perceived impacts of climate change: results from logistic regression analyses ^1^.

Perceived Impacts of Climate Change	Adjusted Odds Ratio (95% Confidence Interval) of Respondents with a High (vs. Low) Level of Understanding on the Causes of Climate Change			
	All	Medical	Public Health	Nursing
Climate change is controllable (yes vs. no)	1.68 **	1.83 **	1.38	1.81
	(1.32–2.14)	(1.29–2.60)	(0.85–2.25)	(1.07–3.06)
Overall, climate change is a bad thing (yes vs. no)	2.16 **	1.98 **	2.33	2.94 **
	(1.57–2.96)	(1.24–3.17)	(1.28–4.24)	(1.44–5.99)
Climate change is bad for human health (yes vs. no)	1.83 **	2.18 **	1.47	2.11
	(1.28–2.60)	(1.27–3.75)	(0.75–2.85)	(1.00–4.50)
Health impacts of climate change will be serious in my local community (yes vs. no)	2.07 **	3.45 **	1.16	2.08
	(1.44–2.99)	(1.93–6.17)	(0.62–2.15)	(0.85–5.07)
Health impacts of climate change will be serious in China (yes vs. no)	5.40 **	9.18 **	2.86	5.55
	(2.60–11.24)	(2.65–31.76)	(0.83–9.87)	(1.12–27.49)
Health impacts of climate change will be serious in the world (yes vs. no)	4.00 **	6.30 **	1.97	4.29
	(2.17–7.39)	(2.47–16.02)	(0.68–5.67)	(0.87–21.17)
Ability to recognize health consequences of climate change (high vs. low)	1.30	1.45	1.27	1.22
	(1.04–1.64)	(1.03–2.04)	(0.83–1.95)	(0.74–2.01)

^1^ Controlled for influences of region, gender, age, discipline (only in the model involving all respondents), income, and health status. ** Bonferroni adjusted *p* < 0.01.

## Supplementary Materials

The following materials are available online at http://www.mdpi.com/1660-4601/15/12/2650/s1. Table S1: Questionnaire items and sources, Table S2: Responses to questions about impacts of climate change, Tables S3–S30: Details of multivariate logistic regression models, Tables S31–S34: Sensitive analysis from a different coding scheme.

## References

[1] Watts N., Adger W.N., Agnolucci P., Blackstock J., Byass P., Cai W., Chaytor S., Colbourn T., Collins M., Cooper A. (2015). Health and climate change: Policy responses to protect public health. Lancet.

[2] Robbins A. (2015). Health consequences of climate change interventions. Lancet.

[3] McIver L., Kim R., Woodward A., Hales S., Spickett J., Katscherian D., Hashizume M., Honda Y., Kim H., Iddings S. (2015). Health Impacts of Climate Change in Pacific Island Countries: A Regional Assessment of Vulnerabilities and Adaptation Priorities. Environ. Health Perspect..

[4] Astrom C., Orru H., Rocklov J., Strandberg G., Ebi K.L., Forsberg B. (2013). Heat-related respiratory hospital admissions in Europe in a changing climate: A health impact assessment. BMJ Open.

[5] Tawatsupa B., Yiengprugsawan V., Kjellstrom T., Seubsman S.A., Sleigh A., Thai Cohort Study Team (2012). Heat stress, health and well-being: Findings from a large national cohort of Thai adults. BMJ Open.

[6] Gill M., Stott R. (2009). Health professionals must act to tackle climate change. Lancet.

[7] Roberts I., Stott R. (2010). Doctors and climate change. Lancet.

[8] Winkler M.S., Roosli M., Ragettli M.S., Cisse G., Muller P., Utzinger J., Perez L. (2015). Mitigating and adapting to climate change: A call to public health professionals. Int. J. Public Health.

[9] Moser A.M., Stigler F.L., Haditsch B. (2017). Physicians’ responsibility for planetary health. Lancet Planet. Health.

[10] Polivka B.J., Chaudry R.V., Mac Crawford J. (2012). Public health nurses’ knowledge and attitudes regarding climate change. Environ. Health Perspect..

[11] Whitley C.T., Takahashi B., Zwickle A., Besley J.C., Lertpratchya A.P. (2018). Sustainability behaviors among college students: An application of the VBN theory. Environ. Educ. Res..

[12] Chaplin G., Wyton P. (2014). Student engagement with sustainability: Understanding the value-action gap. Int. J. Sustain. High. Educ..

[13] Eagle L., Low D., Case P., Vandommele L. (2015). Attitudes of undergraduate business students toward sustainability issues. Int. J. Sustain. High. Educ..

[14] Zsoka A., Szerenyi Z.M., Szechy A., Kocsis T. (2013). Greening due to environmental education? Environmental knowledge, attitudes, consumer behavior and everyday pro-environmental activities of Hungarian high school and university students. J. Clean. Prod..

[15] Prasad V., Thistlethwaite W., Dale W. (2011). Effect of clinical vignettes on senior medical students’ opinions of climate change. South. Med. J..

[16] Ng A.W., Leung T.C.H., Lo J.M.K. (2017). Developing Sustainability Competence for Future Professional Accountants: The Integrative Role of an Undergraduate Program. World Sustain. Ser..

[17] Lambrechts W., Ghijsen P.W.T., Jacques A., Walravens H., Van Liedekerke L., Van Petegem P. (2018). Sustainability segmentation of business students: Toward self-regulated development of critical and interpretational competences in a post-truth era. J. Clean. Prod..

[18] Kagawa F. (2007). Dissonance in students’ perceptions of sustainable development and sustainability: Implications for curriculum change. Int. J. Sustain. High. Educ..

[19] Shi J., Visschers V.H.M., Siegrist M., Arvai J. (2016). Knowledge as a driver of public perceptions about climate change reassessed. Nat. Clim. Chang..

[20] Corner A. (2012). Psychology: Science literacy and climate views. Nat. Clim. Chang..

[21] Shi J., Visschers V.H., Siegrist M. (2015). Public Perception of Climate Change: The Importance of Knowledge and Cultural Worldviews. Risk Anal..

[22] Krosnick J.A., Holbrook A.L., Lowe L., Visser P.S. (2006). The origins and consequences of democratic citizens’ policy agendas: A study of popular concern about global warming. Clim. Chang..

[23] Lorenzoni I., Pidgeon N.F. (2006). Public views on climate change: European and USA perspectives. Clim. Chang..

[24] Bord R.J., O’Connor R.E., Fisher A. (2000). In what sense does the public need to understand global climate change?. Public Underst. Sci..

[25] Tobler C., Visschers V.H.M., Siegrist M. (2012). Consumers’ knowledge about climate change. Clim. Chang..

[26] Maxwell J., Blashki G. (2016). Teaching about Climate Change in Medical Education: An Opportunity. J. Public Health Res..

[27] Friedrich M.J. (2017). Medical Community Gathers Steam to Tackle Climate’s Health Effects. JAMA.

[28] Jing M.A., Yue Y.-G. (2014). Medical Education System in European and American Countries Enlightenments on the Current Medical Education Reform in Our Country. Med. Innov. China.

[29] James H., Handu S.S., Al Khaja K.A.J., Otoom S., Sequeira R.P. (2006). Evaluation of the knowledge, attitude and practice of self-medication among first-year medical students. Med. Princ. Pract..

[30] Johnston J.M., Leung G.M., Fielding R., Tin K.Y.K., Ho L.M. (2003). The development and validation of a knowledge, attitude and behaviour questionnaire to assess undergraduate evidence-based practice teaching and learning. Med. Educ..

[31] Grimm S.R. (2014). Understanding as Knowledge of Causes. Synth Libr..

[32] Hagmayer Y., Witteman C., Ross B.H. (2017). Chapter Four- Causal Knowledge and Reasoning in Decision Making. Psychology of Learning and Motivation.

[33] Lee T.M., Markowitz E.M., Howe P.D., Ko C.Y., Leiserowitz A.A. (2015). Predictors of public climate change awareness and risk perception around the world. Nat. Clim. Chang..

[34] Bedsworth L. (2009). Preparing for climate change: A perspective from local public health officers in California. Environ. Health Perspect..

[35] Wei J., Hansen A., Zhang Y., Li H., Liu Q., Sun Y., Bi P. (2014). Perception, attitude and behavior in relation to climate change: A survey among CDC health professionals in Shanxi province, China. Environ. Res..

[36] Roser-Renouf C., Maibach E.W., Li J. (2016). Adapting to the Changing Climate: An Assessment of Local Health Department Preparations for Climate Change-Related Health Threats, 2008–2012. PLoS ONE.

[37] Maibach E.W., Kreslake J.M., Roser-Renouf C., Rosenthal S., Feinberg G., Leiserowitz A.A. (2015). Do Americans Understand That Global Warming Is Harmful to Human Health? Evidence from a National Survey. Ann. Glob. Health.

[38] McDonald J.H. (2014). Handbook of Biological Statistics.

[39] Hopkinson P., Hughes P., Layer G. (2008). Sustainable graduates: Linking formal, informal and campus curricula to embed education for sustainable development in the student learning experience. Environ. Educ. Res..

[40] Lancet O. (2016). Climate change and non-communicable diseases. Lancet Oncol..

[41] Nigatu A.S., Asamoah B.O., Kloos H. (2014). Knowledge and perceptions about the health impact of climate change among health sciences students in Ethiopia: A cross-sectional study. BMC Public Health.

[42] Duvivier R.J., Watts N.R., Rukavina S., Kaduru C.J. (2011). Doctors talk climate change–students take action. Lancet.

[43] Ding D., Maibach E.W., Zhao X.Q., Roser-Renouf C., Leiserowitz A. (2011). Support for climate policy and societal action are linked to perceptions about scientific agreement. Nat. Clim. Chang..

[44] Green E.I., Blashki G., Berry H.L., Harley D., Horton G., Hall G. (2009). Preparing Australian medical students for climate change. Aust. Fam. Physician.

[45] Rauf S., Bakhsh K., Abbas A., Hassan S., Ali A., Kachele H. (2017). How hard they hit? Perception, adaptation and public health implications of heat waves in urban and peri-urban Pakistan. Environ. Sci. Pollut. Res. Int..

[46] Akompab D.A., Bi P., Williams S., Grant J., Walker I.A., Augoustinos M. (2013). Heat Waves and Climate Change: Applying the Health Belief Model to Identify Predictors of Risk Perception and Adaptive Behaviours in Adelaide, Australia. Int. J. Environ. Res. Public Health.

[47] Kahan D.M., Peters E., Wittlin M., Slovic P., Ouellette L.L., Braman D., Mandel G. (2012). The polarizing impact of science literacy and numeracy on perceived climate change risks. Nat. Clim. Chang..

[48] Bell E.J. (2010). Climate change: What competencies and which medical education and training approaches?. BMC Med. Educ..

[49] Leffers J., Levy R.M., Nicholas P.K., Sweeney C.F. (2017). Mandate for the Nursing Profession to Address Climate Change through Nursing Education. J. Nurs. Scholarsh..

[50] Kreslake J.M., Sarfaty M., Roser-Renouf C., Leiserowitz A.A., Maibach E.W. (2017). The Critical Roles of Health Professionals in Climate Change Prevention and Preparedness. Am. J. Public Health.

[51] Shin G.Y., Manuel R.J. (2016). Healthcare professionals must “think globally, act locally” on climate change. BMJ.

[52] Shaman J., Knowlton K. (2017). The Need for Climate and Health Education. Am. J. Public Health.

[53] Varela-Losada M., Vega-Marcote P., Perez-Rodriguez U., Alvarez-Lires M. (2016). Going to action? A literature review on educational proposals in formal Environmental Education. Environ. Educ. Res..

[54] Mosher H.R., Desrochers M. (2014). The effects of information regarding sustainability issues and behavioral self-management instruction on college students’ energy conservation. Int. J. Sustain. High. Educ..

[55] Lobell D.B., Burke M.B., Tebaldi C., Mastrandrea M.D., Falcon W.P., Naylor R.L. (2008). Prioritizing climate change adaptation needs for food security in 2030. Science.

