# Supplementary material for: Associations between Knowledge of the Causes and Perceived Impacts of Climate Change: A Cross-Sectional Survey of Medical, Public Health and Nursing Students in Universities in China
Source: Int J Environ Res Public Health. 2018 Nov 26;15(12):2650. doi: 10.3390/ijerph15122650 (PMC6313669; doi:10.3390/ijerph15122650)
Supplement: Supplementary file 1 [file ijerph-15-02650-s001.pdf]

Supplementary materials

Table S1 Questionnaire items and sources

<table>
<tr><th colspan="2">Original questions</th><th rowspan="2">Adapted questions in the present study</th></tr>
<tr><th>Questions</th><th>References</th></tr>
<tr><td>In your opinion, extent climate change is:
Controllable
Uncontrollable
Neither uncontrollable nor controllable</td><td>(Polivka et al., 2012)</td><td>Is climate change controllable?
A. Controllable
B. Uncontrollable
C. Neither uncontrollable nor controllable</td></tr>
<tr><td>In general, do you think global warming was a bad or good thing?
A scale from -3 (very bad) to +3 (very good), with no neutral midpoint.</td><td>(Maibach et al., 2015)</td><td>Do you think climate change was a good or bad thing?
"very bad"
"somewhat bad"
"little bad"
"little good"
"somewhat good"
"very good"</td></tr>
<tr><td>Do you think global warming is bad or good for the health of Americans?
A scale from -3 (very bad) to 3 (very good), included “0” as a neutral midpoint.</td><td>(Maibach et al., 2015)</td><td rowspan="2">Do you think climate change is bad or good for human health?
"very bad"
"somewhat bad"
"little bad"
"little good"
"somewhat good"
"very good"</td></tr>
<tr><td>What kind of impacts climate change will bring to us?
None
Positive effects</td><td>(Wei et al., 2014)</td></tr>
</table>

Negative effects
Both of positive effects and negative effects

Cause knowledge of climate change:

Climate change knowledge	Correct	Incorrect	Do not know
1. The global CO_2 concentration in the atmosphere has increased during the past 250 years.			
2. Climate change is mainly caused by human activities.#			
3. The last century's global increase in temperature was the largest during the past 1,000 years.			
4. Today’s global CO_2 concentration in the atmosphere has already occurred in the past 650,000 years. *			

∗Item was reverse coded.

(Shi et al., 2016)

The causes of climate change:

No.	The causes of climate change	Correct	Incorrect	Don’t know
1	Climate change is mainly caused by human activities.			
2	The global CO_2 concentration in the atmosphere has increased during the past 250 years.			
3	The last century's global increase in temperature was the largest during the past 1,000 years.			
4	Today’s global CO_2 concentration in the atmosphere has already occurred in the past 650,000 years. *			

∗Item was reverse coded.

Do you agree that the following issues are the health-related impacts of climate change?

Issues	Yes	No	Don’t know
Vector-borne infectious disease			
Flooding-related displacement			
Mental health conditions			
Air quality-related illness			
Food-borne disease			
Disruption of health care services during extreme weather events			
Water-borne infectious disease			
Heat-related illness			
Cold-related illness			
Water-availability illness			
Malnutrition			
Other climate change health-related impacts			

(Polivka et al., 2012)

Do you agree each following issue as a health impact of climate change?

No.	Issues	Yes	No	Don’t know
1	Vector-borne infectious disease			
2	Flooding-related displacement			
3	Mental health conditions			
4	Air quality-related illness			
5	Food-borne disease			
6	Disruption of health care services during extreme weather events			
7	Water-borne infectious disease			
8	Heat-related illness			
9	Cold-related illness			
10	Water-availability illness			
11	Malnutrition			
12	Other climatic change health-related impacts			

Perception of public health risks associated with climate change:

Items	Very serious	Somewhat serious	Not too serious	Not at all serious

(Bedsworth, 2009)

<table>
<tr>
<td>
<table>
<tr><td>Wildfire</td><td></td><td></td><td></td><td></td></tr>
<tr><td>Extreme weather</td><td></td><td></td><td></td><td></td></tr>
<tr><td>Air pollution</td><td></td><td></td><td></td><td></td></tr>
<tr><td>Heat-related mortality</td><td></td><td></td><td></td><td></td></tr>
<tr><td>Sea-level rise</td><td></td><td></td><td></td><td></td></tr>
<tr><td>Vector-borne illness</td><td></td><td></td><td></td><td></td></tr>
<tr><td>Water contamination</td><td></td><td></td><td></td><td></td></tr>
<tr><td>Water-borne illness</td><td></td><td></td><td></td><td></td></tr>
<tr><td>Food-borne illness</td><td></td><td></td><td></td><td></td></tr>
</table>
</td>
<td></td>
<td></td>
</tr>
<tr>
<td>Do you agree that In the next 20 years, the health-related impacts of climate change will be serious in your jurisdiction?
Agree
Neutral
Disagree
Do you agree that In the next 20 years, the health-related impacts of climate change will be serious in the United States?
Agree
Neutral</td>
<td>(Polivka et al., 2012)</td>
<td>In the next 20 years, if the health-related impacts of climate change would be serious in your community, in China, and globally?
<table>
<tr><th>No.</th><th>In the next 20 years, the health-related impacts of climate change will be serious in:</th><th>strongly agree</th><th>somewhat agree</th><th>mildly agree</th><th>mildly disagree</th><th>somewhat disagree</th><th>strongly disagree</th></tr>
</table>
</td>
</tr>
</table>

<table>
<tr>
<td>Disagree
Do you agree that In the next 20 years, the health-related impacts of climate change will be serious around the world?
Agree
Neutral
Disagree</td>
<td></td>
<td rowspan="2"><table>
<tr><td>1</td><td>My Community</td><td></td><td></td><td></td><td></td><td></td><td></td></tr>
<tr><td>2</td><td>China</td><td></td><td></td><td></td><td></td><td></td><td></td></tr>
<tr><td>3</td><td>Around the world</td><td></td><td></td><td></td><td></td><td></td><td></td></tr>
</table></td>
</tr>
<tr>
<td>Has climate change happened in your local region?
Yes
No
Uncertainty</td>
<td>(Wei et al., 2014)</td>
</tr>
</table>

<table>
<tr>
<td>Using additional variables collected by the Gallup World Poll, we explore the relative influence of individual-level factors in shaping climate change awareness and risk perception among individuals in each nation. These variables include socio-demographics (for example, gender, age, religion, education and location), physical and financial well-being……</td>
<td>(Lee et al., 2015)</td>
<td rowspan="2">Sex:
Male
Female
Age: ________ years old
Grade:
third year
fourth year
fifth year
Major:
Clinical Medicine
Preventive medicine
Nursing
How much is the average monthly income of each family member in your family?
less than 1000
1000-2000
2000-5000
5000-10000
more than 10000
Please assess your health status:
Very good
Good
Medium
Poor</td>
</tr>
<tr>
<td>Responses were analyzed descriptively. Chi-square analysis and analysis of variance determined differences among these respondent subgroups: position type (PHNA, PHN), educational preparation (associate’s degree, baccalaureate, masters/PhD), age (< 54 years, ≥ 55 years), political perspective (moderate, conservative, liberal), and U.S. region (West, Midwest, South, or Northeast).</td>
<td>(Polivka et al., 2012)</td>
</tr>
</table>

References:

Bedsworth, L., 2009. Preparing for climate change: a perspective from local public health officers in California. Environ Health Perspect. 117**,** 617-23.

Lee, T. M., et al., 2015. Predictors of public climate change awareness and risk perception around the world. Nature climate change. 5**,** 1014.

Maibach, E. W., et al., 2015. Do Americans Understand That Global Warming Is Harmful to Human Health? Evidence From a National Survey. Ann Glob Health. 81**,** 396-409.

Polivka, B. J., et al., 2012. Public health nurses' knowledge and attitudes regarding climate change. Environ Health Perspect. 120**,** 321-5.

Shi, J., et al., 2016. Knowledge as a driver of public perceptions about climate change reassessed. 6**,** 759-762.

Wei, J., et al., 2014. Perception, attitude and behavior in relation to climate change: A survey among CDC health professionals in Shanxi province, China. Environmental Research. 134**,** 301-308.

Table S2. Responses to questions about impacts of climate change

	all		Clinical medicine		Preventive medicine		Nursing	
	n	%	n	%	n	%	n	%
**Overall, climate change is bad**								
very bad	241	17.4	126	19.6	61	14.3	54	17.3
somewhat bad	529	38.2	236	36.7	181	42.3	112	35.8
little bad	388	28.0	184	28.6	117	27.3	87	27.8
little good	179	12.9	73	11.4	55	12.9	51	16.3
somewhat good	43	3.1	23	3.6	13	3.0	7	2.2
very good	4	0.3	1	0.2	1	0.2	2	0.6
**Climate change is bad for human health**								
very bad	185	13.4	107	16.7	36	8.4	42	13.6
somewhat bad	597	43.3	263	41.0	198	46.2	136	44.2
little bad	440	31.9	205	31.9	147	34.3	88	28.6
little good	135	9.8	57	8.9	41	9.6	37	12.0
somewhat good	20	1.5	10	1.6	6	1.4	4	1.3
very good	2	0.2	0	0.0	1	0.2	1	0.3
**Climate change will be serious in my local community**								
strongly agree	341	24.6	175	27.2	76	17.7	90	28.9
somewhat agree	484	34.9	224	34.8	146	34.0	114	36.5
little agree	401	28.9	177	27.5	146	34.0	78	25.0
little disagree	89	6.4	38	5.9	34	7.9	17	5.5
somewhat disagree	52	3.8	22	3.4	21	4.9	9	2.9
strongly disagree	19	1.4	8	1.2	7	1.6	4	1.3
**Climate change will be serious in China**								
strongly agree	497	35.9	247	38.4	133	30.9	117	37.5
somewhat agree	560	40.4	246	38.2	183	42.6	131	42.0
little agree	273	19.7	126	19.6	97	22.6	50	16.0
little disagree	43	3.1	19	3.0	13	3.0	11	3.5
somewhat disagree	8	0.6	3	0.5	3	0.7	2	0.6
strongly disagree	5	0.4	3	0.5	1	0.2	1	0.3
**Climate change will be serious in the world**								
strongly agree	542	39.1	257	39.9	150	34.9	135	43.3
somewhat agree	520	37.5	242	37.6	176	40.9	102	32.7
little agree	256	18.5	110	17.1	84	19.5	62	19.9
little disagree	51	3.7	28	4.4	14	3.3	9	2.9
somewhat disagree	11	0.8	4	0.6	4	0.9	3	1.0
strongly disagree	6	0.4	3	0.5	2	0.5	1	0.3

Table S3-S30 Details of multivariate logistic regression models

Independent variable: Pcontrol -- Climate change Controllable or not

Table S3. Multivariate logistic regression analysis on perceptions regarding whether climate change is controllable or not for all medical students

Pcontrol	Odds Ratio	Std. Err.	z	P>z	[95% Conf.	Interval]
**Cause knowledge**	1.69	0.21	4.27	0.00	1.33	2.15
**Gender**	0.80	0.11	-1.62	0.11	0.61	1.05
**Age (ref: ≤20)**						
21-22	1.18	0.25	0.77	0.44	0.78	1.79
>22	1.33	0.35	1.11	0.27	0.80	2.21
**Area (ref: east)**						
North	0.74	0.15	-1.46	0.15	0.50	1.11
Central	0.66	0.14	-1.90	0.06	0.43	1.01
West	0.50	0.11	-3.26	0.00	0.33	0.76
South	0.62	0.13	-2.22	0.03	0.41	0.95
**Major (ref: clinical medicine)**						
Preventive medicine	1.66	0.25	3.37	0.00	1.24	2.23
Nursing	0.95	0.16	-0.33	0.74	0.68	1.31
**Income(ref: < 1000)**						
1000-1999	1.39	0.31	1.47	0.14	0.90	2.16
2000-4999	1.44	0.30	1.77	0.08	0.96	2.17
5000-9999	1.02	0.23	0.09	0.93	0.66	1.57
≥10000	1.06	0.28	0.23	0.82	0.63	1.78
**Health (ref: very good)**						
Good	0.85	0.13	-1.05	0.29	0.63	1.15
Medium or poor	0.80	0.16	-1.13	0.26	0.54	1.18
**_Cons**	1.77	0.65	1.57	0.12	0.87	3.63

Logistic regression — Number of observations = 1311
LR chi2(16) = 61.59
Prob > chi2 = 0.0000
Log likelihood = -795.10691 — Pseudo R2 = 0.0373

Table S4. Multivariate logistic regression analysis on perceptions regarding whether climate change is controllable or not for clinical medicine students

**Pcontrol**	**Odds ratio**	**Std. Err.**	**Z**	**P>z**	**[95% conf.**	**Interval]**
**Cause knowledge**	1.86	0.33	3.46	0.00	1.31	2.63
**Gender**	1.03	0.19	0.14	0.89	0.72	1.46
**Age(ref:≤20)**						
21-22	1.13	0.41	0.33	0.75	0.55	2.30
>22	1.17	0.48	0.39	0.70	0.53	2.60
**Area (ref: east)**						
North	0.44	0.14	-2.56	0.01	0.24	0.83
Central	0.33	0.11	-3.26	0.00	0.17	0.64
West	0.25	0.08	-4.26	0.00	0.13	0.47
South	0.29	0.10	-3.71	0.00	0.15	0.56
**Income(ref: < 1000)**						
1000-1999	1.23	0.42	0.61	0.54	0.63	2.41
2000-4999	1.90	0.60	2.06	0.04	1.03	3.52
5000-9999	1.15	0.38	0.43	0.66	0.60	2.21
≥10000	1.02	0.38	0.04	0.97	0.49	2.10
**Health (ref: very good)**						
Good	0.86	0.19	-0.68	0.49	0.56	1.32
Medium or poor	0.63	0.18	-1.63	0.10	0.37	1.10
**_Cons**	2.66	1.45	1.79	0.07	0.91	7.75

Logistic regression

Number of observations = 619

LR chi2(14) = 50.04

Prob > chi2 = 0.0000

Log likelihood = -377.18229

Pseudo R2 = 0.0622

Table S5. Multivariate logistic regression analysis on perceptions regarding whether climate change is controllable or not for preventive medicine students

**Pcontrol**	**Odds Ratio**	**Std. Err.**	**z**	**P>z**	**[95% Conf.**	**Interval]**
**Cause knowledge**	1.35	0.33	1.23	0.22	0.84	2.19
**Gender**	0.60	0.16	-1.91	0.06	0.36	1.01
**Gage (ref: ≤20)**						
21-22	2.16	1.09	1.52	0.13	0.80	5.82
>22	2.07	1.19	1.26	0.21	0.67	6.37
**Area (ref: East)**						
North	1.31	0.55	0.65	0.52	0.58	2.97
Central	0.85	0.35	-0.40	0.69	0.38	1.88
West	0.91	0.38	-0.24	0.81	0.40	2.07
South	0.99	0.39	-0.02	0.98	0.46	2.13
**Income(ref: < 1000)**						
1000-1999	1.88	0.81	1.46	0.14	0.81	4.35
2000-4999	1.69	0.67	1.34	0.18	0.78	3.67
5000-9999	1.16	0.48	0.36	0.72	0.52	2.62
≥10000	1.08	0.56	0.15	0.88	0.39	2.98
**Health (ref: very good)**						
Good	0.92	0.29	-0.27	0.79	0.49	1.71
Medium or poor	0.75	0.30	-0.73	0.46	0.34	1.63
**_Cons**	1.17	0.85	0.22	0.82	0.29	4.83

Logistic regression

Number of observations = 398
LR chi2(14) = 14.04
Prob > chi2 = 0.4466

Log likelihood = -215.1254

Pseudo R2 = 0.0316

Table S6. Multivariate logistic regression analysis on perceptions regarding whether climate change is controllable or not for nursing students

**Pcontrol**	**Odds Ratio**	**Std. Err.**	**z**	**P>z**	**[95% Conf.**	**Interval]**
Cause knowledge	1.82	0.49	2.25	0.02	1.08	3.07
Gender	0.76	0.31	-0.67	0.51	0.34	1.70
**Age (ref: ≤20)**						
21-22	0.88	0.31	-0.37	0.71	0.44	1.77
>22	1.01	0.53	0.01	0.99	0.36	2.84
**Area (ref: East)**						
North	0.75	0.30	-0.73	0.47	0.34	1.63
Central	1.37	0.60	0.71	0.48	0.58	3.23
West	0.58	0.25	-1.28	0.20	0.25	1.33
South	0.75	0.36	-0.60	0.55	0.29	1.92
**Income (ref: < 1000)**						
1000-1999	1.02	0.46	0.05	0.96	0.42	2.48
2000-4999	0.63	0.27	-1.08	0.28	0.27	1.46
5000-9999	0.54	0.26	-1.27	0.20	0.21	1.39
≥10000	1.02	0.72	0.03	0.97	0.26	4.09
**Health (ref: very good)**						
Good	0.90	0.29	-0.33	0.74	0.48	1.69
Medium or poor	1.65	0.78	1.06	0.29	0.65	4.15
**_Cons**	2.52	1.74	1.34	0.18	0.65	9.73

Logistic regression

Number of observations = 294

LR chi2(14) = 17.84

Prob > chi2 = 0.2142

Log likelihood = -184.39411

Pseudo R2 = 0.0461

Independent variable: Pclimate--Climate change bad or not

Table S7. Multivariate logistic regression analysis on perceptions regarding whether climate change is bad or not for all medical students

Pclimate	Odds Ratio	Std. Err.	z	P>z	[95% Conf.	Interval]
**Cause knowledge**	2.16	0.35	4.81	0.00	1.58	2.96
**Gender**	0.53	0.09	-3.62	0.00	0.38	0.75
**Age(ref: ≤20)**						
21-22	1.66	0.43	1.97	0.05	1.00	2.76
>22	1.52	0.48	1.35	0.18	0.83	2.81
**Area (ref: east)**						
North	1.08	0.25	0.32	0.75	0.68	1.71
Central	1.21	0.31	0.73	0.46	0.73	1.99
West	1.58	0.41	1.74	0.08	0.94	2.63
South	1.75	0.46	2.13	0.03	1.05	2.93
**Major (ref: clinical medicine)**						
Preventive medicine	0.76	0.14	-1.48	0.14	0.52	1.10
Nursing	0.66	0.14	-1.98	0.05	0.43	1.00
**Income (ref: < 1000)**						
1000-1999	1.86	0.51	2.27	0.02	1.09	3.18
2000-4999	1.53	0.37	1.75	0.08	0.95	2.48
5000-9999	1.81	0.49	2.18	0.03	1.06	3.09
≥10000	1.45	0.47	1.16	0.25	0.77	2.73
**Health (ref: very good)**						
Good	1.43	0.26	1.97	0.05	1.00	2.05
Medium or poor	1.65	0.42	1.97	0.05	1.00	2.73
**_Cons**	1.36	0.59	0.72	0.47	0.59	3.17

Logistic regression — Number of observations = 1311
LR chi2(16) = 59.98
Prob > chi2 = 0.0000
Log likelihood = -550.11556 — Pseudo R2 = 0.0517

Table S8. Multivariate logistic regression analysis on perceptions regarding whether climate change is bad or not for Clinical medicine students

**Pclimate**	**Odds Ratio**	**Std. Err.**	**z**	**P>z**	**[95% Conf.**	**Interval]**
**Cause knowledge**	2.00	0.48	2.90	0.00	1.25	3.19
**Gender**	0.55	0.13	-2.49	0.01	0.34	0.88
**Age (ref: ≤20)**						
21-22	1.27	0.62	0.48	0.63	0.49	3.30
>22	0.84	0.44	-0.33	0.74	0.30	2.37
**Area (ref: east)**						
North	0.92	0.32	-0.23	0.82	0.47	1.81
Central	1.20	0.50	0.44	0.66	0.53	2.70
West	1.30	0.51	0.67	0.51	0.60	2.82
South	1.30	0.52	0.67	0.51	0.60	2.84
**Income (ref: < 1000)**						
1000-1999	1.48	0.64	0.92	0.36	0.64	3.44
2000-4999	1.51	0.57	1.08	0.28	0.72	3.18
5000-9999	1.89	0.81	1.48	0.14	0.81	4.37
≥10000	1.28	0.58	0.53	0.59	0.52	3.13
**Health (ref: very good)**						
Good	1.35	0.36	1.12	0.26	0.80	2.29
Medium or poor	1.56	0.59	1.17	0.24	0.74	3.26
**_Cons**	2.50	1.68	1.36	0.18	0.66	9.36

Logistic regression   Number of observations = 619
LR chi2(14) = 26.09
Prob > chi2 = 0.0252
Log likelihood = -247.13464   Pseudo R2 = 0.0501

Table S9. Multivariate logistic regression analysis on perceptions regarding whether climate change is bad or not for Preventive medicine students

**Pclimate**	**Odds Ratio**	**Std. Err.**	**z**	**P>z**	**[95% Conf.**	**Interval]**
**Cause knowledge**	2.31	0.70	2.78	0.01	1.28	4.17
**Gender**	0.63	0.19	-1.51	0.13	0.34	1.15
**Age (ref: ≤20)**						
21-22	1.76	1.05	0.95	0.34	0.55	5.64
>22	2.32	1.56	1.24	0.21	0.62	8.70
**Area (ref: East)**						
North	1.31	0.57	0.61	0.54	0.55	3.10
Central	1.10	0.48	0.21	0.83	0.46	2.59
West	3.27	1.70	2.28	0.02	1.18	9.05
South	4.62	2.40	2.94	0.00	1.67	12.80
**Income (ref: < 1000)**						
1000-1999	2.10	1.09	1.43	0.15	0.76	5.80
2000-4999	1.27	0.59	0.50	0.61	0.51	3.16
5000-9999	1.66	0.86	0.98	0.33	0.60	4.58
≥10000	1.08	0.66	0.13	0.90	0.33	3.58
**Health (ref: very good)**						
Good	1.18	0.44	0.45	0.66	0.57	2.43
Medium or poor	1.33	0.64	0.60	0.55	0.52	3.41
**_Cons**	0.80	0.66	-0.27	0.78	0.16	4.00

Logistic regression — Number of observations = 398

LR chi2(14) = 30.10

Prob > chi2 = 0.0074

Log likelihood = -160.4666 — Pseudo R2 = 0.0858

Table S10. Multivariate logistic regression analysis on perceptions regarding whether climate change is bad or not for Nursing students

Pclimate	Odds Ratio	Std. Err.	z	P>z	[95% Conf.	Interval]
**Cause knowledge**	2.83	1.02	2.88	0.00	1.39	5.74
**Gender**	0.52	0.25	-1.36	0.17	0.20	1.33
**Age (ref: ≤20)**						
21-22	1.22	0.52	0.47	0.64	0.53	2.80
>22	2.17	1.54	1.09	0.28	0.54	8.74
**Area (ref: east)**						
North	1.07	0.54	0.13	0.90	0.40	2.87
Central	1.53	0.86	0.75	0.46	0.50	4.63
West	1.08	0.57	0.14	0.89	0.38	3.05
South	0.89	0.52	-0.19	0.85	0.28	2.80
**Income (ref: ＜1000)**						
1000-1999	2.34	1.25	1.59	0.11	0.82	6.65
2000-4999	1.57	0.77	0.92	0.36	0.60	4.11
5000-9999	1.37	0.75	0.56	0.57	0.46	4.04
≥10000	4.90	5.51	1.41	0.16	0.54	44.47
**Health (ref: very good)**						
Good	2.05	0.75	1.97	0.05	1.01	4.18
Medium or poor	2.68	1.57	1.69	0.09	0.85	8.42
**_Cons**	0.86	0.69	-0.19	0.85	0.18	4.18

Logistic regression Number of observations = 294
LR chi2(14) = 23.44
Prob > chi2 = 0.0534
Log likelihood = -131.4302 Pseudo R2 = 0.0819

Independent variable: Pclihealth--Climate change health impacts bad or not

Table S11. Multivariate logistic regression analysis on perceptions regarding whether climate change health impact is bad or not for all medical students

Pclihealth	Odds Ratio	Std. Err.	z	P>z	[95% Conf.	Interval]
**Cause knowledge**	1.84	0.33	3.37	0.00	1.29	2.61
**Gender**	0.53	0.10	-3.25	0.00	0.36	0.78
**Age (ref: ≤20)**						
21-22	1.60	0.46	1.62	0.11	0.91	2.82
>22	1.74	0.62	1.55	0.12	0.86	3.50
**Area (ref: east)**						
North	1.15	0.30	0.54	0.59	0.69	1.93
Central	1.83	0.56	1.96	0.05	1.00	3.35
West	1.36	0.39	1.07	0.29	0.78	2.37
South	1.53	0.44	1.47	0.14	0.87	2.68
**Major (ref: clinical medicine)**						
Preventive medicine	0.80	0.17	-1.02	0.31	0.53	1.22
Nursing	0.64	0.15	-1.88	0.06	0.40	1.02
**Income (ref: ＜1000)**						
1000-1999	1.17	0.35	0.52	0.61	0.65	2.11
2000-4999	1.48	0.42	1.39	0.16	0.85	2.59
5000-9999	1.34	0.41	0.95	0.34	0.73	2.45
≥10000	1.36	0.51	0.83	0.41	0.65	2.85
**Health (ref: very good)**						
Good	1.19	0.25	0.86	0.39	0.80	1.79
Medium or poor	1.51	0.45	1.39	0.16	0.85	2.69
**_Cons**	2.75	1.34	2.07	0.04	1.06	7.15

Logistic regression	Number of observations =	1311
	LR chi2(16)=	34.69
	Prob > chi2 =	0.0044
Log likelihood = -457.03837	Pseudo R2 =	0.0366

Table S12. Multivariate logistic regression analysis on perceptions regarding whether climate change health impact is bad or not for clinical medicine students

**Pclihealth**	**Odds Ratio**	**Std. Err.**	**z**	**P>z**	**[95% Conf.**	**Interval]**
**Cause knowledge**	2.19	0.60	2.85	0.00	1.28	3.77
**Gender**	0.52	0.15	-2.30	0.02	0.30	0.91
**Age (ref: ≤20)**						
21-22	1.47	0.78	0.73	0.46	0.52	4.16
>22	1.17	0.69	0.27	0.79	0.37	3.70
**Area (ref: east)**						
North	1.03	0.43	0.07	0.94	0.45	2.35
Central	1.43	0.73	0.70	0.48	0.53	3.89
West	0.77	0.33	-0.61	0.54	0.33	1.80
South	0.95	0.42	-0.13	0.90	0.39	2.28
**Income (ref: ＜1000)**						
1000-1999	0.98	0.48	-0.04	0.96	0.37	2.58
2000-4999	1.25	0.57	0.49	0.62	0.51	3.06
5000-9999	1.07	0.52	0.13	0.89	0.41	2.80
≥10000	0.97	0.52	-0.06	0.95	0.34	2.78
**Health (ref: very good)**						
Good	1.19	0.37	0.55	0.58	0.65	2.18
Medium or poor	1.31	0.56	0.63	0.53	0.57	3.02
**_Cons**	4.99	3.82	2.10	0.04	1.11	22.37

Logistic regression	Number of observations	=	619
	LR chi2(14)	=	17.87
	Prob > chi2	=	0.2127
Log likelihood = -201.15144	Pseudo R2	=	0.0425

Table S13. Multivariate logistic regression analysis on perceptions regarding whether climate change health impact is bad or not for preventive medicine students

**Pclihealth**	**Odds Ratio**	**Std. Err.**	**z**	**P>z**	**[95% Conf.**	**Interval]**
**Cause knowledge**	1.47	0.49	1.13	0.26	0.76	2.84
**Gender**	0.58	0.20	-1.58	0.11	0.29	1.14
**Age (ref: ≤20)**						
21-22	2.29	1.47	1.29	0.20	0.65	8.06
>22	3.19	2.35	1.58	0.12	0.75	13.53
**Area (ref: east)**						
North	1.05	0.49	0.10	0.92	0.42	2.63
Central	1.53	0.78	0.84	0.40	0.57	4.15
West	3.36	2.03	2.01	0.05	1.03	10.96
South	4.48	2.64	2.54	0.01	1.41	14.22
**Income (ref: ＜1000)**						
1000-1999	1.46	0.85	0.66	0.51	0.47	4.58
2000-4999	1.09	0.58	0.17	0.87	0.39	3.07
5000-9999	1.39	0.81	0.56	0.57	0.44	4.37
≥10000	1.07	0.77	0.09	0.93	0.26	4.41
**Health (ref: very good)**						
Good	1.31	0.53	0.66	0.51	0.59	2.89
Medium or poor	1.84	1.05	1.08	0.28	0.61	5.60
**_Cons**	1.21	1.08	0.21	0.84	0.21	7.00

Logistic regression	Number of observations	=	398
	LR chi2(14)	=	19.15
	Prob > chi2	=	0.1594
Log likelihood = -130.87121	Pseudo R2	=	0.0682

Table S14. Multivariate logistic regression analysis on perceptions regarding whether climate change health impact is bad or not for nursing students

Pclihealth	Odds Ratio	Std. Err.	z	P>z	[95% Conf.	Interval]
**Cause knowledge**	2.10	0.81	1.93	0.05	1.00	4.48
**Gender**	0.76	0.40	-0.53	0.60	0.27	2.13
**Age (ref: ≤20)**						
21-22	0.94	0.45	-0.12	0.90	0.37	2.41
>22	1.55	1.24	0.54	0.59	0.32	7.45
**Area (ref: east)**						
North	1.27	0.67	0.46	0.65	0.46	3.55
Central	5.34	4.40	2.03	0.04	1.06	26.85
West	1.27	0.71	0.43	0.67	0.42	3.79
South	0.86	0.51	-0.26	0.80	0.26	2.78
**Income (ref: ＜1000)**						
1000-1999	0.98	0.54	-0.03	0.98	0.33	2.89
2000-4999	1.72	0.97	0.96	0.34	0.57	5.21
5000-9999	1.03	0.63	0.05	0.96	0.31	3.44
≥10000	2.96	3.39	0.95	0.34	0.32	27.84
**Health (ref: very good)**						
Good	1.37	0.57	0.76	0.45	0.61	3.09
Medium or poor	2.11	1.37	1.14	0.25	0.59	7.55
**_Cons**	2.14	1.89	0.86	0.39	0.38	12.14

Logistic regression	Number of observations	=	294
	LR chi2(14)	=	17.75
	Prob > chi2	=	0.2186
Log likelihood = -113.47916	Pseudo R2	=	0.0725

Independent variable: Local0--Future threats in local

Table S15. Multivariate logistic regression analysis on health threats of climate change health impact is serious or not in future in local community for all medical students

**Local0**	**Odds Ratio**	**Std. Err.**	**z**	**P>z**	**[95% Conf.**	**Interval]**
**Cause knowledge**	2.07	0.39	3.90	0.00	1.44	2.98
**Gender**	0.68	0.14	-1.90	0.06	0.46	1.01
**Age (ref: ≤20)**						
21-22	2.17	0.61	2.76	0.01	1.25	3.75
>22	2.26	0.80	2.30	0.02	1.13	4.53
**Area (ref: East)**						
North	1.61	0.50	1.52	0.13	0.87	2.96
Central	1.35	0.43	0.96	0.34	0.73	2.51
West	0.78	0.22	-0.86	0.39	0.44	1.37
South	1.05	0.31	0.16	0.87	0.59	1.86
**Major (ref: Clinical medicine)**						
Preventive medicine	0.73	0.15	-1.53	0.13	0.48	1.09
Nursing	1.18	0.31	0.63	0.53	0.70	1.98
**Income (ref: ＜1000)**						
1000-1999	0.81	0.26	-0.65	0.52	0.43	1.53
2000-4999	1.02	0.32	0.06	0.96	0.55	1.87
5000-9999	1.03	0.35	0.10	0.92	0.53	2.01
≥10000	1.11	0.45	0.27	0.79	0.50	2.47
**Health (ref: very good)**						
Good	0.81	0.19	-0.90	0.37	0.52	1.28
Medium or poor	0.80	0.23	-0.78	0.44	0.45	1.42
**_Cons**	3.91	1.99	2.68	0.01	1.44	10.60

Logistic regression — Number of observations = 1311

LR chi2(16) = 38.59

Prob > chi2 = 0.0012

Log likelihood = -442.85598 — Pseudo R2 = 0.0417

Table S16. Multivariate logistic regression analysis on health threats of climate change health impact is serious or not in future in local community for clinical medicine students

**Local0**	**Odds Ratio**	**Std. Err.**	**z**	**P>z**	**[95% Conf.**	**Interval]**
**Cause knowledge**	3.40	1.01	4.14	0.00	1.90	6.07
**Gender**	0.69	0.19	-1.34	0.18	0.40	1.19
**Age (ref: ≤20)**						
21-22	1.51	0.76	0.82	0.41	0.57	4.03
>22	1.75	1.01	0.96	0.34	0.56	5.44
**Area (ref: East)**						
North	1.44	0.63	0.83	0.41	0.61	3.40
Central	2.10	1.11	1.40	0.16	0.74	5.94
West	0.96	0.42	-0.10	0.92	0.40	2.26
South	0.93	0.41	-0.17	0.86	0.39	2.19
**Income (ref: ＜1000)**						
1000-1999	0.48	0.25	-1.38	0.17	0.17	1.36
2000-4999	0.67	0.33	-0.80	0.42	0.25	1.78
5000-9999	1.18	0.68	0.30	0.77	0.39	3.63
≥10000	1.02	0.64	0.03	0.97	0.30	3.48
**Health (ref: very good)**						
Good	1.14	0.36	0.41	0.68	0.61	2.12
Medium or poor	1.92	0.89	1.41	0.16	0.77	4.76
**_Cons**	3.89	3.03	1.74	0.08	0.84	17.94

Logistic regression	Number of observations	=	619
	LR chi2(14)	=	32.69
	Prob > chi2	=	0.0032
Log likelihood = -191.60666	Pseudo R2	=	0.0786

Table S17. Multivariate logistic regression analysis on health threats of climate change health impact is serious or not in future in local community for preventive medicine students

**Local0**	**Odds Ratio**	**Std. Err.**	**z**	**P>z**	**[95% Conf.**	**Interval]**
**Cause knowledge**	1.16	0.37	0.47	0.64	0.62	2.16
**Gender**	0.71	0.24	-1.01	0.32	0.37	1.38
**Age (ref: ≤20)**						
21-22	1.71	1.07	0.86	0.39	0.50	5.81
>22	1.33	0.95	0.40	0.69	0.33	5.38
**Area (ref: East)**						
North	1.81	1.09	0.98	0.33	0.56	5.90
Central	0.61	0.31	-0.98	0.33	0.22	1.64
West	0.48	0.25	-1.41	0.16	0.17	1.33
South	1.28	0.69	0.46	0.64	0.45	3.70
**Income (ref: ＜1000)**						
1000-1999	2.01	1.13	1.24	0.21	0.67	6.05
2000-4999	1.55	0.78	0.87	0.39	0.58	4.17
5000-9999	1.49	0.83	0.72	0.47	0.50	4.44
≥10000	0.96	0.62	-0.06	0.96	0.27	3.40
**Health (ref: very good)**						
Good	0.50	0.24	-1.45	0.15	0.20	1.28
Medium or poor	0.37	0.20	-1.82	0.07	0.12	1.08
**_Cons**	6.42	6.11	1.95	0.05	0.99	41.44

Logistic regression — Number of observations = 398
LR chi2(14) = 16.67
Prob > chi2 = 0.2743
Log likelihood = -147.82545 — Pseudo R2 = 0.0534

Table S18. Multivariate logistic regression analysis on health threats of climate change health impact is serious or not in future in local community for nursing students

**Local0**	**Odds Ratio**	**Std. Err.**	**z**	**P>z**	**[95% Conf.**	**Interval]**
**Cause knowledge**	2.08	0.95	1.61	0.11	0.85	5.07
**Gender**	1.00	0.70	0.00	1.00	0.25	3.96
**Age (ref: ≤20)**						
21-22	2.40	1.25	1.68	0.09	0.87	6.64
>22	2.93	2.74	1.15	0.25	0.47	18.36
**Area (ref: East)**						
North	2.06	1.51	0.99	0.32	0.49	8.66
Central	2.91	2.57	1.21	0.23	0.51	16.46
West	0.64	0.44	-0.64	0.52	0.17	2.46
South	0.72	0.53	-0.45	0.65	0.17	3.04
**Income (ref: ＜1000)**						
1000-1999	0.57	0.42	-0.76	0.45	0.14	2.41
2000-4999	1.11	0.83	0.13	0.89	0.25	4.83
5000-9999	0.36	0.28	-1.33	0.18	0.08	1.63
≥10000	1.00	(empty)				
**Health (ref: very good)**						
Good	0.66	0.37	-0.75	0.45	0.22	1.97
Medium or poor	0.40	0.28	-1.32	0.19	0.10	1.57
**_Cons**	7.14	7.97	1.76	0.08	0.80	63.67

Logistic regression	Number of observations	=	280
	LR chi2(13)	=	21.63
	Prob > chi2	=	0.0614
Log likelihood = -84.526992	Pseudo R2	=	0.1134

Independent variable: China0--Future threats in China

Table S19. Multivariate logistic regression analysis on health threats of climate change health impact is serious or not in future in China for all medical students

China0	Odds Ratio	Std. Err.	z	P>z	[95% Conf.	Interval]
**Cause knowledge**	5.44	2.03	4.53	0.00	2.61	11.31
**Gender**	0.57	0.19	-1.72	0.09	0.30	1.08
**Age (ref: ≤20)**						
21-22	1.34	0.59	0.66	0.51	0.57	3.16
>22	2.44	1.49	1.46	0.15	0.73	8.10
**Area (ref: East)**						
North	1.73	0.91	1.04	0.30	0.62	4.85
Central	1.11	0.55	0.20	0.84	0.42	2.93
West	1.01	0.48	0.03	0.98	0.40	2.59
South	0.97	0.46	-0.07	0.94	0.38	2.43
**Major (ref: Clinical medicine)**						
Preventive medicine	0.97	0.34	-0.08	0.94	0.49	1.94
Nursing	0.81	0.32	-0.54	0.59	0.38	1.74
**Income (ref: ＜1000)**						
1000-1999	1.51	0.69	0.91	0.37	0.62	3.69
2000-4999	1.96	0.83	1.59	0.11	0.85	4.51
5000-9999	1.85	0.87	1.30	0.19	0.73	4.67
≥10000	2.19	1.36	1.26	0.21	0.65	7.40
**Health (ref: very good)**						
Good	1.26	0.42	0.69	0.49	0.66	2.41
Medium or poor	1.68	0.81	1.08	0.28	0.66	4.30
**_Cons**	5.31	4.04	2.19	0.03	1.19	23.63

Logistic regression — Number of observations = 1311
LR chi2(16) = 39.97
Prob > chi2 = 0.0008
Log likelihood = -205.12425 — Pseudo R2 = 0.0888

Table S20. Multivariate logistic regression analysis on health threats of climate change health impact is serious or not in future in China for clinical medicine students

China0	**Odds Ratio**	**Std. Err.**	**z**	**P>z**	**[95% Conf.**	**Interval]**
**Cause knowledge**	9.25	5.86	3.51	0.00	2.67	32.00
**Gender**	0.83	0.37	-0.41	0.68	0.35	1.98
**Age (ref: ≤20)**						
21-22	0.50	0.54	-0.65	0.52	0.06	4.08
>22	0.67	0.79	-0.34	0.74	0.07	6.79
**Area (ref: East)**						
North	1.32	0.98	0.38	0.71	0.31	5.66
Central	1.28	1.07	0.29	0.77	0.25	6.59
West	0.70	0.50	-0.50	0.62	0.17	2.82
South	0.73	0.53	-0.44	0.66	0.18	3.00
**Income (ref: ＜1000)**						
1000-1999	0.83	0.59	-0.26	0.80	0.21	3.31
2000-4999	1.49	0.99	0.60	0.55	0.41	5.47
5000-9999	1.73	1.31	0.73	0.47	0.39	7.63
≥10000	1.66	1.53	0.55	0.58	0.28	10.05
**Health (ref: very good)**						
Good	1.96	0.93	1.41	0.16	0.77	4.97
Medium or poor	2.90	2.05	1.51	0.13	0.73	11.56
**_Cons**	11.95	16.13	1.84	0.07	0.85	168.55

Logistic regression	Number of observations =	619
	LR chi2(14)=	25.95
	Prob > chi2 =	0.0263
Log likelihood = -88.555634	Pseudo R2 =	0.1278

Table S21. Multivariate logistic regression analysis on health threats of climate change health impact is serious or not in future in China for preventive medicine students

**China0**	**Odds Ratio**	**Std. Err.**	**z**	**P>z**	**[95% Conf.**	**Interval]**
**Cause knowledge**	2.85	1.77	1.69	0.09	0.84	9.61
**Gender**	0.51	0.30	-1.16	0.25	0.16	1.59
**Age (ref: ≤20)**						
21-22	1.13	1.27	0.11	0.92	0.12	10.28
>22	4.60	7.10	0.99	0.32	0.22	95.01
**Area (ref: East)**						
North	1.08	1.15	0.08	0.94	0.13	8.71
Central	0.51	0.49	-0.70	0.49	0.08	3.41
West	0.60	0.61	-0.50	0.61	0.08	4.32
South	1.60	1.73	0.43	0.67	0.19	13.35
**Income (ref: ＜1000)**						
1000-1999	2.36	2.12	0.95	0.34	0.40	13.74
2000-4999	3.40	2.99	1.39	0.17	0.60	19.12
5000-9999	3.99	4.04	1.37	0.17	0.55	28.99
≥10000	2.58	2.74	0.89	0.37	0.32	20.73
**Health (ref: very good)**						
Good	1.28	0.85	0.37	0.71	0.35	4.69
Medium or poor	1.20	1.00	0.22	0.83	0.23	6.19
**_Cons**	6.29	9.77	1.19	0.24	0.30	131.77

Logistic regression	Number of observations	=	398
	LR chi2(14)	=	12.58
	Prob > chi2	=	0.5599
Log likelihood = -57.600281	Pseudo R2	=	0.0984

Table S22. Multivariate logistic regression analysis on health threats of climate change health impact is serious or not in future in China for nursing students

**China0**	**Odds Ratio**	**Std. Err.**	**z**	**P>z**	**[95% Conf.**	**Interval]**
**Cause knowledge**	5.50	4.49	2.09	0.04	1.11	27.23
**Gender**	0.25	0.20	-1.70	0.09	0.05	1.23
**Age (ref: ≤20)**						
21-22	1.73	1.28	0.74	0.46	0.40	7.39
>22	4.40	6.19	1.05	0.29	0.28	69.21
**Area (ref: East)**						
North	4.33	5.27	1.20	0.23	0.40	46.98
Central	1.27	1.26	0.24	0.81	0.18	8.84
West	2.44	2.60	0.84	0.40	0.30	19.76
South	0.62	0.60	-0.49	0.62	0.09	4.12
**Income (ref: ＜1000)**						
1000-1999	2.74	2.71	1.02	0.31	0.40	19.04
2000-4999	1.58	1.34	0.54	0.59	0.30	8.31
5000-9999	1.08	0.99	0.09	0.93	0.18	6.54
≥10000	1.00	(empty)				
**Health (ref: very good)**						
Good	0.37	0.31	-1.20	0.23	0.07	1.88
Medium or poor	1.26	1.69	0.17	0.86	0.09	17.36
**_Cons**	9.35	14.23	1.47	0.14	0.47	184.65

Logistic regression	Number of observations	=	280
	LR chi2(13)	=	19.71
	Prob > chi2	=	0.1026
Log likelihood = -48.635918	Pseudo R2	=	0.1685

Independent variable: World0--Future threats in world

Table S23. Multivariate logistic regression analysis on health threats of climate change health impact is serious or not in future around the world for all medical students

World0	Odds Ratio	Std. Err.	z	P>z	[95% Conf.	Interval]
**Cause knowledge**	4.01	1.25	4.44	0.00	2.17	7.40
**Gender**	0.59	0.17	-1.82	0.07	0.33	1.04
**Age (ref: ≤20)**						
21-22	1.45	0.60	0.91	0.37	0.65	3.27
>22	2.48	1.39	1.62	0.10	0.83	7.45
**Area (ref: East)**						
North	1.23	0.56	0.45	0.65	0.50	3.02
Central	1.14	0.54	0.28	0.78	0.45	2.89
West	1.01	0.45	0.02	0.98	0.42	2.44
South	0.95	0.42	-0.10	0.92	0.40	2.28
**Major (ref: Clinical medicine)**						
Preventive medicine	1.00	0.31	0.00	1.00	0.54	1.84
Nursing	1.19	0.45	0.47	0.64	0.57	2.51
**Income (ref: ＜1000)**						
1000-1999	1.49	0.68	0.88	0.38	0.61	3.64
2000-4999	1.59	0.66	1.13	0.26	0.71	3.57
5000-9999	1.53	0.69	0.93	0.35	0.63	3.71
≥10000	0.95	0.47	-0.10	0.92	0.36	2.51
**Health (ref: very good)**						
Good	1.73	0.52	1.85	0.07	0.97	3.11
Medium or poor	1.63	0.66	1.20	0.23	0.73	3.62
**_Cons**	4.23	2.99	2.05	0.04	1.06	16.86

Logistic regression	Number of observations	=	1311
	LR chi2(16)	=	37.74
	Prob > chi2	=	0.0017
Log likelihood = -236.80214	Pseudo R2	=	0.0738

Table S24. Multivariate logistic regression analysis on health threats of climate change health impact is serious or not in future around the world for clinical medicine students

World0	Odds Ratio	Std. Err.	z	P>z	[95% Conf.	Interval]
**Cause knowledge**	6.36	3.03	3.88	0.00	2.50	16.18
**Gender**	0.59	0.24	-1.31	0.19	0.27	1.29
**Age (ref: ≤20)**						
21-22	1.22	0.84	0.29	0.78	0.31	4.73
>22	2.10	1.75	0.89	0.37	0.41	10.72
**Area (ref: East)**						
North	1.07	0.67	0.11	0.91	0.31	3.65
Central	2.16	1.72	0.97	0.33	0.45	10.29
West	0.72	0.45	-0.53	0.60	0.21	2.44
South	0.96	0.64	-0.06	0.95	0.26	3.53
**Income (ref: ＜1000)**						
1000-1999	1.35	1.03	0.39	0.69	0.30	6.01
2000-4999	0.86	0.54	-0.25	0.81	0.25	2.95
5000-9999	1.31	0.95	0.37	0.71	0.31	5.46
≥10000	0.58	0.42	-0.75	0.46	0.14	2.39
**Health (ref: very good)**						
Good	2.18	0.88	1.94	0.05	0.99	4.81
Medium or poor	5.88	4.63	2.25	0.03	1.26	27.50
**_Cons**	5.00	5.21	1.54	0.12	0.65	38.50

Logistic regression — Number of observations = 619

LR chi2(14)= 34.48

Prob > chi2 = 0.0018

Log likelihood = -108.71587 — Pseudo R2 = 0.1369

Table S25. Multivariate logistic regression analysis on health threats of climate change health impact is serious or not in future around the world for preventive medicine students

**World0**	**Odds Ratio**	**Std. Err.**	**z**	**P>z**	**[95% Conf.**	**Interval]**
**Cause knowledge**	1.98	1.06	1.27	0.21	0.69	5.68
**Gender**	0.56	0.30	-1.09	0.28	0.20	1.58
**Age (ref: ≤20)**						
21-22	0.80	0.89	-0.20	0.84	0.09	7.21
>22	5.33	8.18	1.09	0.28	0.26	107.93
**Area (ref: East)**						
North	1.40	1.24	0.38	0.71	0.25	7.99
Central	1.04	0.85	0.05	0.96	0.21	5.18
West	1.79	1.67	0.62	0.53	0.29	11.11
South	1.58	1.33	0.54	0.59	0.30	8.20
**Income (ref: ＜1000)**						
1000-1999	1.10	0.88	0.12	0.90	0.23	5.24
2000-4999	5.69	5.61	1.76	0.08	0.82	39.28
5000-9999	1.60	1.35	0.56	0.58	0.31	8.33
≥10000	1.06	0.99	0.06	0.95	0.17	6.61
**Health (ref: very good)**						
Good	1.29	0.85	0.38	0.70	0.35	4.67
Medium or poor	0.46	0.32	-1.11	0.27	0.12	1.83
**_Cons**	8.58	12.82	1.44	0.15	0.46	160.38

Logistic regression	Number of observations	=	398
	LR chi2(14)	=	19.92
	Prob > chi2	=	0.1327
Log likelihood = -66.376882	Pseudo R2	=	0.1305

Table S26. Multivariate logistic regression analysis on health threats of climate change health impact is serious or not in future around the world for nursing students

World0	Odds Ratio	Std. Err.	z	P>z	[95% Conf.	Interval]
**Cause knowledge**	4.23	3.43	1.77	0.08	0.86	20.77
**Gender**	0.58	0.52	-0.60	0.55	0.10	3.39
**Age (ref: ≤20)**						
21-22	1.25	1.01	0.27	0.79	0.26	6.09
>22	0.55	0.67	-0.49	0.63	0.05	6.11
**Area (ref: East)**						
North	1.86	2.43	0.48	0.63	0.14	24.08
Central	0.76	0.83	-0.25	0.81	0.09	6.47
West	0.80	0.94	-0.19	0.85	0.08	7.99
South	0.24	0.28	-1.23	0.22	0.02	2.36
**Income (ref: ＜1000)**						
1000-1999	5.36	6.44	1.40	0.16	0.51	56.51
2000-4999	1.26	1.06	0.27	0.79	0.24	6.60
5000-9999	1.17	1.12	0.16	0.87	0.18	7.65
≥10000	1.00	(empty)				
**Health (ref: very good)**						
Good	1.07	0.79	0.09	0.93	0.25	4.57
Medium or poor	2.18	2.70	0.63	0.53	0.19	24.51
**_Cons**	13.48	21.75	1.61	0.11	0.57	318.27

Logistic regression	Number of observations	=	280
	LR chi2(13)	=	14.41
	Prob > chi2	=	0.3453
Log likelihood = -45.394183	Pseudo R2	=	0.1370

Independent variable: Gtcliheath--Identification health impacts of climate change

Table S27. Multivariate logistic regression analysis on identification of climate change health impact is high or not for all medical students

Gtcliheath	Odds Ratio	Std. Err.	z	P>z	[95% Conf.	Interval]
**Cause knowledge**	1.31	0.15	2.29	0.02	1.04	1.64
**Gender**	0.84	0.11	-1.34	0.18	0.65	1.09
**Age (ref: ≤20)**						
21-22	1.27	0.27	1.14	0.25	0.84	1.91
>22	1.22	0.30	0.79	0.43	0.75	1.98
**Area (ref: East)**						
North	1.34	0.25	1.59	0.11	0.93	1.94
Central	1.18	0.23	0.85	0.39	0.80	1.74
West	0.91	0.18	-0.49	0.62	0.62	1.33
South	1.24	0.24	1.09	0.28	0.84	1.81
**Major (ref: Clinical medicine)**						
Preventive medicine	0.72	0.10	-2.35	0.02	0.55	0.95
Nursing	0.66	0.11	-2.55	0.01	0.48	0.91
**Income (ref: ＜1000)**						
1000-1999	0.79	0.17	-1.09	0.28	0.52	1.21
2000-4999	1.05	0.21	0.25	0.80	0.71	1.56
5000-9999	1.07	0.23	0.31	0.76	0.70	1.63
≥10000	1.41	0.37	1.30	0.19	0.84	2.37
**Health (ref: very good)**						
Good	0.66	0.10	-2.76	0.01	0.49	0.89
Medium or poor	0.54	0.10	-3.25	0.00	0.37	0.78
**_Cons**	1.50	0.53	1.16	0.25	0.76	3.00

Logistic regression	Number of observations	=	1268
	LR chi2(16)	=	44.32
	Prob > chi2	=	0.0002
Log likelihood = -845.31829	Pseudo R2	=	0.0255

Table S28. Multivariate logistic regression analysis on identification of climate change health impact is high or not for clinical medicine students

**Gtcliheath**	**Odds Ratio**	**Std. Err.**	**z**	**P>z**	**[95% Conf.**	**Interval]**
**Cause knowledge**	1.46	0.25	2.16	0.03	1.04	2.05
**Gender**	0.81	0.14	-1.18	0.24	0.57	1.15
**Age (ref: ≤20)**						
21-22	1.28	0.46	0.67	0.50	0.63	2.60
>22	1.02	0.41	0.05	0.96	0.46	2.24
**Area (ref: East)**						
North	1.60	0.44	1.72	0.09	0.94	2.74
Central	1.46	0.45	1.22	0.22	0.79	2.69
West	0.88	0.25	-0.44	0.66	0.50	1.55
South	1.31	0.38	0.91	0.36	0.74	2.31
**Income (ref: ＜1000)**						
1000-1999	0.74	0.26	-0.88	0.38	0.37	1.46
2000-4999	0.93	0.30	-0.22	0.82	0.50	1.74
5000-9999	0.88	0.30	-0.36	0.72	0.45	1.72
≥10000	1.24	0.47	0.55	0.58	0.58	2.62
**Health (ref: very good)**						
Good	0.78	0.17	-1.19	0.24	0.51	1.18
Medium or poor	0.87	0.24	-0.52	0.61	0.50	1.49
**_Cons**	1.31	0.70	0.50	0.62	0.46	3.71

Logistic regression	Number of observations =	596
	LR chi2(14)=	19.35
	Prob > chi2 =	0.1519
Log likelihood = -386.84117	Pseudo R2 =	0.0244

Table S29. Multivariate logistic regression analysis on identification of climate change health impact is high or not for preventive medicine students

Gtcliheath	Odds Ratio	Std. Err.	z	P>z	[95% Conf.	Interval]
**Cause knowledge**	1.27	0.28	1.10	0.27	0.83	1.95
**Gender**	0.94	0.23	-0.24	0.81	0.58	1.53
**Age (ref: ≤20)**						
21-22	2.16	1.13	1.48	0.14	0.78	6.01
>22	1.87	1.07	1.09	0.28	0.61	5.73
**Area (ref: East)**						
North	1.15	0.41	0.38	0.70	0.57	2.31
Central	0.68	0.24	-1.09	0.27	0.34	1.36
West	0.69	0.26	-1.00	0.32	0.33	1.43
South	1.22	0.42	0.58	0.56	0.62	2.40
**Income (ref: ＜1000)**						
1000-1999	1.07	0.41	0.17	0.86	0.51	2.25
2000-4999	1.42	0.50	0.99	0.33	0.71	2.83
5000-9999	1.50	0.57	1.07	0.29	0.71	3.17
≥10000	2.32	1.15	1.70	0.09	0.88	6.12
**Health (ref: very good)**						
Good	0.58	0.17	-1.88	0.06	0.33	1.02
Medium or poor	0.38	0.14	-2.67	0.01	0.18	0.77
**_Cons**	0.70	0.48	-0.52	0.60	0.18	2.72

Logistic regression	Number of observations	=	385
	LR chi2(14)	=	21.49
	Prob > chi2	=	0.0896
Log likelihood = -255.16758	Pseudo R2	=	0.0404

Table S30. Multivariate logistic regression analysis on identification of climate change health impact is high or not for nursing students

Gtcliheath	Odds Ratio	Std. Err.	z	P>z	[95% Conf.	Interval]
**Cause knowledge**	1.22	0.31	0.78	0.44	0.74	2.01
**Gender**	1.05	0.42	0.12	0.91	0.47	2.32
**Age (ref: ≤20)**						
21-22	0.73	0.26	-0.90	0.37	0.36	1.45
>22	1.47	0.76	0.74	0.46	0.53	4.06
**Area (ref: East)**						
North	1.24	0.48	0.56	0.58	0.58	2.66
Central	1.67	0.70	1.22	0.22	0.73	3.78
West	1.11	0.46	0.25	0.81	0.49	2.51
South	0.77	0.36	-0.56	0.57	0.31	1.92
**Income (ref: ＜1000)**						
1000-1999	0.72	0.31	-0.76	0.45	0.31	1.68
2000-4999	0.84	0.35	-0.43	0.67	0.37	1.89
5000-9999	0.86	0.40	-0.32	0.75	0.34	2.15
≥10000	1.15	0.77	0.21	0.84	0.31	4.29
**Health (ref: very good)**						
Good	0.59	0.19	-1.66	0.10	0.31	1.10
Medium or poor	0.35	0.16	-2.37	0.02	0.15	0.84
**_Cons**	1.94	1.31	0.98	0.33	0.52	7.31

Logistic regression	Number of observations	=	287
	LR chi2(14)	=	15.27
	Prob > chi2	=	0.3600
Log likelihood = -191.28258	Pseudo R2	=	0.0384

Table S31-S34: Sensitive analysis for codification methods

Cause knowledge: Using the summed scores 0-4 for the knowledge of causes regarding climate change

Table S31. Multivariate logistic regression analysis on identification of climate change health impact is high or not for all medical students

Gtcliheath	Odds Ratio	Std. Err.	z	P>z	[95% Conf.	Interval]
**Cause knowledge**	1.20	0.07	3.13	0.00	1.07	1.34
**Gender**	0.84	0.11	-1.32	0.19	0.65	1.09
**Age (ref: ≤20)**						
21-22	1.26	0.26	1.12	0.26	0.84	1.91
>22	1.22	0.30	0.80	0.43	0.75	1.99
**Area (ref: East)**						
North	1.38	0.26	1.72	0.09	0.96	1.99
Central	1.22	0.24	1.01	0.32	0.83	1.80
West	0.93	0.18	-0.36	0.72	0.63	1.37
South	1.26	0.25	1.17	0.24	0.86	1.84
**Income (ref: ＜1000)**						
1000-1999	0.71	0.10	-2.43	0.02	0.54	0.94
2000-4999	0.66	0.11	-2.55	0.01	0.48	0.91
5000-9999						
≥10000	0.78	0.17	-1.16	0.25	0.51	1.19
**Health (ref: very good)**	1.04	0.21	0.18	0.86	0.70	1.54
Good	1.07	0.23	0.30	0.76	0.70	1.63
Medium or poor	1.42	0.37	1.32	0.19	0.84	2.38
**_Cons**						
2	0.66	0.10	-2.79	0.01	0.49	0.88
3	0.54	0.10	-3.22	0.00	0.37	0.78
**_Cons**	1.13	0.42	0.32	0.75	0.54	2.34

Logistic regression   Number of observations = 1268
LR chi2(16) = 48.89
Prob > chi2 = 0.0000
Log likelihood = -843.03472   Pseudo R2 = 0.0282

Table S32. Multivariate logistic regression analysis on identification of climate change health impact is high or not for clinical medicine students

Gtcliheath	Odds Ratio	Std. Err.	z	P>z	[95% Conf.	Interval]
**Cause knowledge**	1.25	0.11	2.66	0.01	1.06	1.48
**Gender**	0.81	0.14	-1.15	0.25	0.58	1.15
**Age (ref: ≤20)**						
21-22	1.27	0.46	0.66	0.51	0.62	2.60
>22	1.03	0.41	0.08	0.94	0.47	2.27
**Area (ref: East)**						
North	1.68	0.46	1.88	0.06	0.98	2.87
Central	1.57	0.49	1.45	0.15	0.85	2.91
West	0.92	0.27	-0.28	0.78	0.52	1.62
South	1.34	0.39	1.00	0.32	0.76	2.38
**Income (ref: ＜1000)**						
1000-1999	0.73	0.25	-0.90	0.37	0.37	1.45
2000-4999	0.92	0.29	-0.27	0.78	0.49	1.71
5000-9999	0.88	0.30	-0.37	0.71	0.45	1.72
≥10000	1.23	0.47	0.55	0.58	0.58	2.62
**Health (ref: very good)**						
Good	0.76	0.16	-1.25	0.21	0.50	1.16
Medium or poor	0.86	0.24	-0.55	0.58	0.50	1.48
**_Cons**	0.92	0.52	-0.15	0.88	0.31	2.76

Logistic regression Number of observations = 596
LR chi2(14) = 21.78
Prob > chi2 = 0.0833
Log likelihood = -385.62948 Pseudo R2 = 0.0275

Table S33. Multivariate logistic regression analysis on identification of climate change health impact is high or not for preventive medicine students

Gtcliheath	Odds Ratio	Std. Err.	z	P>z	[95% Conf.	Interval]
**Cause knowledge**	1.18	0.13	1.52	0.13	0.95	1.46
**Gender**	0.95	0.24	-0.20	0.84	0.59	1.55
**Age (ref: ≤20)**						
21-22	2.15	1.12	1.48	0.14	0.78	5.95
>22	1.88	1.07	1.11	0.27	0.61	5.75
**Area (ref: East)**						
North	1.15	0.41	0.39	0.70	0.57	2.31
Central	0.68	0.25	-1.06	0.29	0.34	1.38
West	0.71	0.26	-0.92	0.36	0.34	1.47
South	1.25	0.43	0.64	0.52	0.63	2.45
**Income (ref: ＜1000)**						
1000-1999	1.06	0.40	0.14	0.89	0.50	2.22
2000-4999	1.41	0.50	0.96	0.34	0.70	2.81
5000-9999	1.53	0.58	1.11	0.27	0.72	3.24
≥10000	2.40	1.19	1.76	0.08	0.91	6.37
**Health (ref: very good)**						
Good	0.58	0.17	-1.91	0.06	0.33	1.01
Medium or poor	0.39	0.14	-2.58	0.01	0.19	0.80
**_Cons**	0.52	0.39	-0.88	0.38	0.12	2.23

Logistic regression	Number of observations	=	385
	LR chi2(14)	=	22.60
	Prob > chi2	=	0.0671
Log likelihood = -254.61314	Pseudo R2	=	0.0425

Table S34. Multivariate logistic regression analysis on identification of climate change health impact is high or not for nursing students

Gtcliheath	Odds Ratio	Std. Err.	z	P>z	[95% Conf.	Interval]
**Cause knowledge**	1.18	0.15	1.31	0.19	0.92	1.51
**Gender**	1.02	0.41	0.06	0.95	0.46	2.27
**Age (ref: ≤20)**						
21-22	0.72	0.25	-0.94	0.35	0.36	1.43
>22	1.46	0.76	0.72	0.47	0.53	4.03
**Area (ref: East)**						
North	1.27	0.50	0.61	0.54	0.59	2.74
Central	1.68	0.70	1.23	0.22	0.74	3.81
West	1.11	0.46	0.25	0.81	0.49	2.51
South	0.77	0.36	-0.56	0.58	0.31	1.93
**Income (ref: ＜1000)**						
1000-1999	0.71	0.31	-0.79	0.43	0.31	1.66
2000-4999	0.83	0.34	-0.46	0.65	0.37	1.87
5000-9999	0.86	0.40	-0.34	0.74	0.34	2.14
≥10000	1.13	0.76	0.19	0.85	0.30	4.24
**Health (ref: very good)**						
Good	0.59	0.19	-1.63	0.10	0.32	1.11
Medium or poor	0.34	0.15	-2.42	0.02	0.14	0.82
**_Cons**	1.49	1.08	0.56	0.58	0.36	6.13

Logistic regression	Number of observations	=	287
	LR chi2(14)	=	16.40
	Prob > chi2	=	0.2896
Log likelihood = -190.7178	Pseudo R2	=	0.0412

We also performed sensitivity tests by introducing the summed score (0-4) of causes knowledge into the regression models.
The sensitivity tests produced consistent results although the AORs changed.
